# Response of Kapok seedlings were irrigated with water of different qualities and heavy metal contents for foliar application of antioxidants

**DOI:** 10.1186/s12870-024-05902-y

**Published:** 2025-01-03

**Authors:** Alaa I. B. Abou-Sreea, Faisal M. A. Matter, Mahmoud A. Hassanain, Abdallah H. A. Hassan

**Affiliations:** Horticulture Department, Faculty of Agriculture, Fayoum University, Fayoum, 63514 Egypt

**Keywords:** Kapok, Sewage water, Agricultural drainage water, Glycine betaine (GB), *Ceiba pentandra*, Water scarcity

## Abstract

**Background:**

The imbalance between Egypt's water requirements and supply necessitates the use of unconventional water sources, such as treated sewage water (TSW) and agricultural drainage water (ADW), to combat water scarcity. This study investigated the effects of foliar glycine betaine (GB) on vegetative growth parameters, physiological characteristics, photosynthetic pigments, leaf element contents, anatomical leaf structures, and antioxidant activity. The experiment was conducted in two successive seasons (2021/2022 and 2022/2023) using Kapok seedlings irrigated with ADW and TSW at different mixing ratios with normal irrigation water (NIW) (25%, 50%, 75%, and 100%), combined with foliar spraying of GB at concentrations of 0.0 and 50 mM.

**Results:**

The results revealed that irrigation with 100% TSW or ADW significantly decreased vegetative growth parameters, physiological characteristics, photosynthetic pigments, leaf element contents, leaf thickness, and the contents of the leaf mid-vein, N, P, K, and Ca. In contrast, the levels of free proline, total phenolic content, Na, Cu, Ni, Mn, Zn, Pb, and antioxidant activity increased. Additionally, GB significantly improved all parameters, while reducing the contents of Na, Cu, Ni, Mn, Zn, and Pb in the leaves.

**Conclusions:**

Irrigation of Kapok seedlings with TSW or ADW mixed with NIW at 25% and 50% resulted in better performance, similar to irrigation with NIW alone for most parameters. Combining GB and water treatments by mixing TSW or ADW with NIW at a 50:50 ratio and spraying with 50 mM GB produced better results than control seedlings irrigated with 100% NIW. Antioxidants also play a defensive role in plants against various stress factors. Therefore, GB may have a protective effect on peroxidation-linked membrane deterioration, scavenge free radicals, and provide osmotic protection.

**Supplementary Information:**

The online version contains supplementary material available at 10.1186/s12870-024-05902-y.

## Introduction

Kapok tree or silk cotton tree (*Ceiba pentandra*,) Fam. Malvaceae, a promising woody tree, has several medicinal and nutritional properties [1]. The fibers of kapok trees have been used locally as fiberfill in quilts, pillows, and some soft toys as well as in pulp and paper manufacturing [2, 3]. *Ceiba pentandra* L. can reach a height of 50 m in tropical America, Asia, and Africa, this plant is well utilized in traditional medicine in Africa for the treatment of several diseases, such as headache, constipation, dizziness, mental troubles, fever and as a diuretic [4, 5].

Global water consumption has increased by 1% annually since the 1980s due to population growth, socioeconomic development, urbanization, climate change, and changing water use patterns. By 2050, water demand is expected to grow by 20–30%, driven mainly by industrial and domestic use, though agricultural use will still be the largest category [6]. Egypt is severely affected by climate change within and outside its borders in the Nile Basin. Decreasing River Nile levels, population growth, water contamination, traditional irrigation systems, and persistent droughts contribute to water scarcity. Since 2000, Egypt has been below the water poverty line, requiring new water resources by 2050 [7, 8] and the water sector is the most exposed to this change [9, 10]. The Nile River supplies about 97% of Egypt's freshwater, with an annual share fixed at 55.5 billion cubic meters. This is insufficient to meet growing water demands, prompting Egypt to enhance wastewater reuse strategies since 2004, approved in 2005 (Egyptian Code 501/2005) [11]. Egypt's imbalance between water requirements and supply necessitates using unconventional sources like recycled water and groundwater. Recycling agricultural drainage water (ADW) has reached its limit, making further increases challenging [12]. Water quality issues in Egypt stem from varied usage patterns and an increasing population. Climate-induced droughts and protected farming further challenge stable agricultural water supply. Wastewater reuse for agriculture is gaining international attention as an alternative water source and is widely applied globally [13].

Glycine betaine (GB) (N, N, N-trimethyl glycine) is amphoteric (able to react both as a base and as an acid.) compounds that are electrically neutral over a wide range of physiological pH values. Glycine betaine (GB) is soluble in water but contains a nonpolar hydrocarbon moiety that is composed of three methyl groups. Glycine betaine (GB) molecular features permit it to interact with both domains of macromolecules (hydrophobic and hydrophilic), such as enzymes and protein complexes. Studies in vitro have shown that Glycine betaine (GB) is a nontoxic, cellular osmolyte that increases intracellular osmolarity if a cell is subjected to stress-induced hyperosmotic: conditions Glycine betaine (GB) has been well reported to stabilize enzyme and protein complex structures and activities in vitro and conserves the integrity of membranes against the negative effects of severe salt, heat, and cold [14].

This study aims to investigate the effects of using treated sewage water and agricultural drainage water for irrigation of forest trees, compared with those of Nile irrigation water, on the growth biomass, physiological parameters, and chemical constituents of Kapok seedlings to increase the amount of water income from the available resources in Egypt.

## Materials and methods

### Experimental description

Kapok seeds were taken from local trees, in Fayoum, Egypt, during the season (2019/2020). First, the Kapok seeds were sown in a plastic pot (10 cm in top diameter) filled with 2 kg of sand and clay soil (3:1 v/v) in the first week of April for both seasons. Then, the seedlings were transplanted 90 days after sowing into the plastic pot (20 cm in top diameter) in the same soil and irrigated with NIW. Second, one year after sowing for both seasons, the seedlings were cultivated in plastic pots (40 cm top diameter) filled with 20 kg of sandy soil and each bag contained one seedling (the seedlings height 30–40 cm), The experiment was conducted during two agricultural seasons in 2021–2022 and 2022–2023. The experiment began in both seasons in April and ended in September for a period of six months for each season. The Physical and chemical properties of the experimental soil were determined according to the methods and procedures outlined and described previously [15, 16]. The obtained results are tabulated in Table 1.

**Table 1:** Physical and chemical properties of the experimental soil in the two seasons of 2021 and 2022

Properties **SI & SII**								
Physical properties								
Clay (%)			Silt (%)		Sand (%)		Soil texture	
SI	5.10		5.60		89.30		sandy	
SII	4.90		5.10		90.61		sandy	
Chemical properties								
ECe (ds/m/25 °C)					pH			
SI	2.39				7.12			
SII	2.07				7.06			
Soluble cations (meq l^−1^)					Soluble anions (meq l^−1^)			
Na^+^		Mg^2+^	Na^+^	Mg^2+^	Na^+^	Mg^2+^	Na^+^	SO4^=^
SI	13.40	7.50	3.00	0.36	6.86	0.00	10.80	6.24
SII	13.90	6.80	2.80	0.29	6.03	0.01	9.90	5.92
Available macronutrients (mg/ kg soil)					Available micronutrients (mg/ kg soil)			
N		P	K		Fe	Zn	Cu	Mn
SI	4.25	4.45	108.00		2.85	0.07	0.385	20.80
SII	4.25	4.01	110.00		2.71	0.06	0.368	19.40

The study aims to investigate the effects of using treated sewage water and agricultural drainage water for irrigation of forest trees, compared with those of Nile irrigation water, on the growth biomass, physiological parameters, and chemical constituents of Kapok seedlings, to increase the amount of water income from the available resources in Egypt.

### Water treatments

Different Types of water (agricultural drainage water, treated sewage water, and Nile irrigation water) were obtained from different resources: treated sewage water was obtained from a bilateral sewage treatment plant in the new city of Beni Suef, and the water analyses are shown in Table 2. Agricultural drainage water was obtained from agricultural irrigation water canals in the village of Naim Beni suef, and the analyses are shown in Table 2. Nile irrigation water was obtained from irrigation canals in the village of Naim Beni suef. The different experiments were carried out in combination with agricultural drainage water and Nile irrigation water at ratios of 0:100, 25:75, 50:50, 75:25 and 100:0%. Additionally, treated sewage water and Nile irrigation water were combined at ratios of 0:100, 25:75, 50:50, 75:25, and 100:0%, respectively, with various concentrations of the antioxidant foliar Glycine betaine (GB) (0 and 50 mM).

**Table 2:** Analysis of treated sewage water (TSW) and agricultural drainage water (ADW) used in this study during the two seasons of 2021 (SI) and 2022 (SII)

Properties	Treated sewage water	Agricultural drainage water	Treated sewage water	Agricultural drainage water
	**SI**		**SII**	
**ECe (ds/m at 25 °C)**	6.19	1.15	6.15	1.11
**pH (at 25°C)**	7.19	7.34	7.14	7.41
**Na (meq/L) soluble Na**	54.10	5.50	53.90	5.62
**Ca + Mg (meq/L)**	14. 00	6. 00	14.20	6.11
**SAR (Sodium Adsorption Ratio)**	20.50	3.18	20.30	3.22
**Fe (mg/L)**	18.86	11.08	18.69	11.23
**Zn (mg/L)**	4.10	6.32	4.13	6.39
**Cu (mg/L)**	0.39	0.42	0.36	0.47
**Mn (mg/L)**	0.13	0.59	0.14	0.62
**Pb (mg/L)**	6.54	5.88	6.49	5.63
**Ni (mg/L)**	2.61	5.20	2.58	5.31
**Na (mg/L)**	2185.62	1511.44	2162.11	1539.72

### Glycine betaine (GB) application

After the seedlings stabilized, they were transplanted 45 days after they were transferred and irrigated with concentrations of different water mixing ratios (water treatments). The seedlings were sprayed with Glycine betaine (GB) at different concentrations (0.0 and 50 mM) at 75, 105, and 135 days after they were transferred Glycine betaine (GB) was obtained from Sigma.

### Experimental layout

The experimental design used was a split plot in randomized complete blocks with three replications. The main plots consisted of the application water quality (9 treatments), whereas the sub-plots were allocated to the Glycine betaine (GB) concentrations (2 treatments). All the treatments (18 treatments) had three replications, with each replication containing ten plastic bags; each treatment consisted of thirteen plastic bags.

### Data recorded

All vegetative, physiological and chemical traits were estimated for both growing seasons after six months of irrigating the seedlings with different water types.

### Vegetative growth characteristics

Vegetative growth characteristics were estimated in both seasons as follows: Plant height (cm) was measured in the main stem starting from the ground level to the apical meristem. The plant leaf number is mathematically calculated by evaluating the net number of leaves surrounding the main stem of Kapok seedlings. The plant stem diameter (mm) was measured by using a Sealy So707-Digital Electronic Vernier Caliper 150 mm/6–60 above ground by 5 cm. the fresh weights (g) of the stems, leaves, and roots were mathematically calculated from the fresh weight of each plant organ. The root diameter (mm) was measured by using a Sealy So707-Digital Electronic Vernier Caliper 150 mm/6–0״ underground by 2 cm. Stem, leaf, and root (g) dry weights were obtained by drying at 70°C in a forced-air oven until the weight became constant. The leaf area index (m^2^) is mathematically calculated via the leaf area leaf weight relationship as illustrated [17]. The root length (cm) was measured starting from the ground level to the apical meristem of the main root.

### Physiological parameters

The index of membrane stability (MSI %) was evaluated using a 0.2 g sample of fully expanded leaf tissue. The MSI was calculated via the following formula [18]. MSI (%) = [1-(C1⁄C2) × 100. At full flowering, the relative water content (RWC %) was analyzed in three leaf samples from the third node from the shoot top (three replications per treatment). Leaf discs (*n* = 20; diameter = 5 mm) were cut from each leaf. The relative water content was calculated via the following equation [18]. The relative chlorophyll content (SPAD value) was estimated from one plant leaf/plant to determine the SPAD chlorophyll index via a chlorophyll meter (SPAD- 502, Minolta, Japan).

### Chemical compositions

#### Leaf photosynthetic pigments

The contents of leaf chlorophyll (a, b, and carotenoid concentrations) (mg mm^2^ fresh weight) were calculated and estimated according to [19]. Leaf samples (0.1 g) were ground in a porcelain mortar by using 25 ml of 80% (v∕v) acetone. After filtration, the absorbance of each acetone extract was estimated at 663, 645, and 470 nm by using a UV-160A UV–visible recording spectrometer (Shimadzu, Kyoto, Japan).

#### Leaf elemental contents

In each experimental unit, leaf samples from three randomly selected plants, were collected, washed with tap water, rinsed three times with distilled water, and dried at 70°c in a forced-air oven until a constant weight was reached. The N (%) of each leaf was calorimetrically determined via the orange G dye according to the methods of [20]. P (mg g-1 dry weight) of the Leaves was calorimetrically measured according to the standard molybdate chloride method described previously [21]. Leaf K + , Ca, and Na + (mg g^−1^ dry weight) contents were photometrically estimated via a flam photometer as described previously [22]. Leaf Cu and Ni (ppm) and Mn, Pb and Zn (mg/100 g dry weight) were photometrically measured via atomic absorption spectroscopy.

#### Leaf osmoprotectant compounds and antioxidant activity

Free proline (mg g^−1^ dry weight) was measured by using ninhydrin reagent as described previously [23]. The total phenolic content in the plant (mg g^−1^ dry weight) was extracted and calculated according to the Folin Ciocalteu colorimetric method described by [24] and modified by [25]. DPPH radical scavenging capacity was measured via the 1, 1-diphenyl-2-picrylhydrazyl (DPPH) assay [26] which was applied with some alterations.

### Anatomical study

Agricultural drainage water and Nile irrigation water were applied at (ratios of 0:100, 25: 75, 50:50, 75:25, and 100:0), respectively, in combination with treated sewage water and Nile irrigation water at (ratios of 0:100%, 25: 75%, 50:50%, 75:25% and 100: 0%), respectively, with concentrations of the antioxidant foliar Glycine betaine (GB) (0 and 50 mM). Kapok plant samples were obtained from the fully expanded leaf (4th leaf from the growing tip of the plants, including the mid-rip), at 1 cm in length at 65 days of age. The slides were microscopically analyzed, and the sections were micro photographed according to [27].

### Statistical analysis

Data from both experimental seasons were analyzed using GenStat statistical software (version 11, VSN International Ltd., Oxford, UK). Means comparisons among treatments were performed using Duncan’s Multiple Range Test at a significance level of *p* ≤ 0.05 [28].

## Results

### Growth parameters

Notably irrigation with TSW and ADW caused a remarkable decrease in and damage to all vegetative growth parameters (Tables 3, 4, and 5). There was a significant decrease in the height of the plant; the number of leaves on the plant; the stem diameter/plant, stem, leaf and root fresh weights; the root diameter; the stem; leaf and root dry weights; the leaf area index; and the root length, which were negatively affected by irrigation at 100% (TSW or ADW). On the other hand, when TSW or ADW was mixed with NIW at a percentage of 50:50 positive results were obtained for all the growth, periods. Similarly, control seedlings were irrigated with NIW.

**Table 3:** Main and interaction effects of water treatments (WT) and glycine betaine (GB) foliar application on plant height (cm), number of leaves, root length (cm) and stem diameter of Kapok seedlings during 2021 (SI) and 2022 (SII) seasons

Water treatments (WT)	Glycine betaine (GB)	Plant height (cm)		leaves number/plant		Root length (cm)		Stem diameter (mm)	
		**SI**	**SII**	**SI**	**SII**	**SI**	**SII**	**SI**	**SII**
**NIW**		**90.33 A**	**91.00 A**	**20.33 A**	**20.50 A**	**65.00 A**	**64.67 A**	**20.33 A**	**20.50 A**
**ADW 1**		**86.50 A**	**86.33 AB**	**20.17 A**	**19.83 AB**	**62.50 A**	**62.66 A**	**20.17 A**	**21.00 A**
**ADW 2**		**80.50 B**	**82.33 BC**	**18.19 A**	**18.32 AB**	**59.83 A**	**61.17 A**	**19.67 A**	**19.67 A**
**ADW 3**		**67.50 C**	**71.83 D**	**12.35 B**	**13.83 C**	**50.50 B**	**48.83 B**	**13.83 B**	**15.00 B**
**ADW 4**		**64.00 C**	**58.50 E**	**10.00 C**	**10.84 D**	**46.83 B**	**38.68 B**	**11.67 CD**	**11.50 CD**
**TSW 1**		**85.33 AB**	**82.17 BC**	**19.32 A**	**19.00 AB**	**65.17 A**	**64.17 A**	**20.17 A**	**19.33 A**
**TSW 2**		**80.00 B**	**78.00 CD**	**18.15 A**	**17.50 B**	**62.50 A**	**61.50 A**	**19.33 A**	**19.50 A**
**TSW 3**		**66.00 C**	**63.50 E**	**9.83 C**	**10.66 D**	**45.17 BC**	**41.67 A**	**13.50 BC**	**13.00 C**
**TSW 4**		**47.83 D**	**46.17 F**	**8.19 C**	**9.67 D**	**40.00 C**	**40.18 B**	**11.33 D**	**10.50 D**
	**0mM**	**71.11 B**	**69.00 B**	**14.75 B**	**14.32 B**	**53.00 B**	**50.00 B**	**15.44 B**	**15.37 B**
	**50mM**	**79.00 A**	**77.63 A**	**16.22 A**	**16.81 A**	**58.00 A**	**57.40 A**	**17.89 A**	**17.96 A**
NIW	0mM	88.33 ab	88.67 abc	19.29 ab	19.68 ab	61.33 a-d	60.00 a	19.00 abc	19.00 abc
	50mM	92.33 a	93.33 a	21.34 a	21.29 a	68.67 ab	69.32 a	21.67 a	22.00 a
ADW 1	0mM	83.00 bc	82.67 bcd	20.04 a	19.34 ab	60.33 a-d	60.68 a	19.33 abc	20.00 ab
	50mM	90.00 ab	90.00 ab	20.09 a	20.31 ab	64.67 abc	64.65 a	21.00 ab	22.00 a
ADW 2	0mM	75.67 cd	79.33 cde	16.28 bc	17.00 bc	55.00 c-f	57.33 ab	18.33 bc	18.67 bc
	50mM	85.33 ab	85.33 a-d	20.11 a	19.65 ab	64.67 abc	65.00 a	21.00 ab	20.66 ab
ADW 3	0mM	62.67 f	71.33 e	10.05 de	12.00 de	49.33 efg	41.32 c	12.33 fg	13.34 ef
	50mM	72.33 de	72.33 e	14.67 c	15.61 cd	51.67 d-g	56.34 ab	15.33 de	16.68 cd
ADW 4	0mM	62.33 f	56.67 f	9.00 de	10.00 ef	46.33 fgh	36.68 c	11.00g	10.33 fg
	50mM	65.67 ef	60.33 f	11.31 d	11.58 ef	47.33 fgh	40.64 c	12.33 fg	12.69 ef
TSW 1	0mM	82.00 bc	78.00 de	18.00 ab	18.33 abc	61.00 a-d	59.69 a	19.00 abc	18.32 bc
	50mM	88.67 ab	86.33 a-d	21.34 a	19.62 ab	69.33 a	68.67 a	21.33 ab	20.31 ab
TSW 2	0mM	74.00 d	71.00 e	16.29 bc	15.43 cd	58.33 b-e	57.34 ab	17.67 cd	18.00 bc
	50mM	86.00 ab	85.00 a-d	20.00 a	19.57 ab	66.67 ab	65.69 a	21.00 ab	21.00 ab
TSW 3	0mM	58.33 f	54.67 f	10.05 de	8.77 f	45.67 fgh	39.67 c	12.33 fg	11.33 fg
	50mM	73.67 d	72.33 e	10.02 de	12.57 de	44.67 gh	43.66 bc	14.67 ef	14.67 de
TSW 4	0mM	35.00 g	38.67 g	7.28 e	8.58 f	38.00 h	37.00 c	10.00 g	9.31 g
	50mM	60.67 f	53.67 f	9.64 de	10.69 ef	42.00 gh	43.00 bc	12.67 efg	11.69 efg

*NIW* Nile irrigation water 100%, *ADW1* agricultural drainage water 25%:Nile irrigation water 75%, *ADW2* agricultural drainage water 50%: Nile irrigation water 50%, *ADW3* agricultural drainage water 75%:Nile irrigation water 25%, *ADW4* agricultural drainage water100%, *TSW1* treated sewage water 25%:Nile irrigation water 75%, *TSW2* treated sewage water 50%:Nile irrigation water 50%, *TSW3* treated sewage water 25%: Nile irrigation water 75%, *TSW4* treated sewage water 100%

Values marked with the same letter(s) within the main and interaction impacts are statistically similar using Duncan’s multiple range test. Uppercase letter(s) refers to differences within the main effects and lowercase letter(s) refers to differences within the interaction effects

**Table 4:** Main and interaction effects of water treatments (WT) and glycine betaine (GB) foliar application on leaf area index (m^2^), root diameter (mm), stem fresh weight and fresh weight of leaves of Kapok seedlings during 2021 (SI) and 2022 (SII) seasons

Water treatments (WT)	Glycine betaine (GB)	Leaf area index (m2)		Root diameter (mm)		Stem fresh weight (g)		Leaves fresh weight (g)	
		**SI**	**SII**	**SI**	**SII**	**SI**	**SII**	**SI**	**SII**
**NIW**		**0.0884 A**	**0.0887 A**	**19.67 A**	**19.67 A**	**94.11 A**	**92.18 A**	**23.04 A**	**22.97 A**
**ADW 1**		**0.0879 A**	**0.0877 A**	**19.33AB**	**19.50 A**	**88.99 AB**	**87.42 A**	**21.88 A**	**22.16 A**
**ADW 2**		**0.0877 A**	**0.0879 A**	**17.33AB**	**18.17 A**	**83.62 B**	**86.18 A**	**20.93 A**	**21.76 A**
**ADW 3**		**0.0831 B**	**0.0827 B**	**14.33 C**	**13.83 B**	**50.33 C**	**50.46 B**	**16.47 B**	**15.03 B**
**ADW 4**		**0.0821 B**	**0.0817 BC**	**14.33 C**	**13.83 B**	**39.13 D**	**35.08 C**	**12.90 BC**	**13.48 B**
**TSW 1**		**0.0879 A**	**0.0883 A**	**19.33AB**	**18.83 A**	**89.84AB**	**88.24 A**	**21.71 A**	**23.31 A**
**TSW 2**		**0.0871 A**	**0.0876 A**	**17.83AB**	**18.00 A**	**85.75AB**	**85.17 A**	**22.61 A**	**21.75 A**
**TSW 3**		**0.0817 BC**	**0.0823 B**	**13.67CD**	**13.83 B**	**37.64 D**	**37.85 C**	**14.78 BC**	**14.56 B**
**TSW 4**		**0.0803 C**	**0.0805 C**	**11.67 D**	**11.50 B**	**27.53 E**	**25.22 D**	**11.80 C**	**11.73 B**
	**0mM**	**0.0844 B**	**0.0843 B**	**15.4 B**	**15.40 B**	**60.31 B**	**59.43 B**	**16.44 B**	**16.44 B**
	**50mM**	**0.0859 A**	**0.0862 A**	**17.4 A**	**17.30 A**	**72.35 A**	**71.19 A**	**20.47 A**	**20.61 A**
NIW	0mM	0.0877 a	0.0888 a	19.33 ab	19.00 abc	92.02 ab	88.57 abc	20.51 abc	20.37 bc
	50mM	0.0890 a	0.0886 a	20.00 a	20.33 a	96.21 a	95.78 a	25.58 a	25.57 a
ADW 1	0mM	0.0871 a	0.0870 a	18.33 abc	18.33 a-d	82.85 bc	80.53 c	19.82 a-d	20.31 bc
	50mM	0.0887 a	0.0884 a	20.33 a	20.67 a	95.14 ab	94.31 ab	23.93 ab	24.01 ab
ADW 2	0mM	0.0875 a	0.0872 a	16.33 b-e	17.67 a-d	73.18 c	79.87 c	19.26 bcd	19.39 bcd
	50mM	0.0878 a	0.0886 a	18.33 a-d	18.67 a-d	94.06 ab	92.50 ab	22.60 abc	24.13 ab
ADW 3	0mM	0.0827 bc	0.0819 bc	13.00 e-i	14.00 e–h	41.96 efg	42.98 e	14.57 d-g	12.80 efg
	50mM	0.0835 b	0.0834 b	15.67 c-g	13.67 e–h	58.70 d	57.94 d	18.38 b-e	17.26 cde
ADW 4	0mM	0.0809 cde	0.0805 c	13.33 e-i	12.33 fgh	34.52 fgh	28.91 f	12.29 fg	12.11 eg
	50mM	0.0833 b	0.0829 bc	15.33 c-h	15.33 d-g	43.75 ef	41.24 e	13.51 efg	14.85 d-g
TSW 1	0mM	0.0877 a	0.0880 a	18.33 abc	18.33 a-d	86.18 ab	83.59 bc	18.30 b-e	20.17 bc
	50mM	0.0880 a	0.0886 a	20.33 a	19.33 ab	93.51 ab	92.89 ab	25.13 a	26.45 a
TSW 2	0mM	0.0870 a	0.0874 a	16.33 b-e	16.34 b-e	83.27 bc	80.98 c	19.98 a-d	19.71 bcd
	50mM	0.0872 a	0.0878 a	19.33 ab	19.68 ab	88.24 ab	89.36 abc	25.25 a	23.79 ab
TSW 3	0mM	0.0795 de	0.0809 c	12.33 hi	12.00 gh	25.28 h	25.29 f	12.49 fg	11.94 g
	50mM	0.0838 b	0.0837 b	15.00 c-h	15.66 c-f	50.00 de	50.41 de	17.07 c-f	17.18 c-f
TSW 4	0mM	0.0790 e	0.0772 d	11.00 i	10.68 h	23.52 h	24.17 f	10.77 g	11.19 g
	50mM	0.0816 bcd	0.0837 b	12.33 hi	12.34 fgh	31.53 gh	26.26 f	12.82 efg	12.26 efg

*NIW* Nile irrigation water 100%, *ADW1* agricultural drainage water 25%:Nile irrigation water 75%, *ADW2* agricultural drainage water 50%: Nile irrigation water 50%, *ADW3* agricultural drainage water 75%:Nile irrigation water 25%, *ADW4* agricultural drainage water100%, *TSW1* treated sewage water 25%:Nile irrigation water 75%, *TSW2* treated sewage water 50%:Nile irrigation water 50%, *TSW3* treated sewage water 25%: Nile irrigation water 75%, *TSW4* treated sewage water 100%

Values marked with the same letter(s) within the main and interaction impacts are statistically similar using Duncan's multiple range test. Uppercase letter(s) refers to differences within the main effects and lowercase letter(s) refers to differences within the interaction effects

**Table 5:** Main and interaction effects of water treatments (WT) and glycine betaine (GB) foliar application on fresh weight of roots (g), stem dry weight (g), leaves dry weight (g) and roots dry weight (g) of Kapok seedlings during 2021 (SI) and 2022 (SII) seasons

Water treatments (WT)	Glycine betaine (GB)	Roots fresh weight (g)		Stem dry weight (g)		Leaves dry weight (g)		Roots dry weight (g)	
		**SI**	**SII**	**SI**	**SII**	**SI**	**SII**	**SI**	**SII**
**NIW**		**87.19 A**	**87.96 A**	**30.31 A**	**28.99 A**	**6.703 A**	**6.712 A**	**28.90 A**	**28.10 A**
**ADW 1**		**83.31 A**	**82.45 A**	**28.25 AB**	**27.92 AB**	**6.503 A**	**6.535 AB**	**27.63 A**	**27.28 A**
**ADW 2**		**83.77 A**	**84.12 A**	**25.91 BC**	**25.19 BC**	**6.040 AB**	**6.280 AB**	**26.10 A**	**25.73 A**
**ADW 3**		**55.02 B**	**53.10 B**	**13.24 D**	**12.89 DE**	**4.150 C**	**4.210 C**	**18.89 B**	**15.86 BC**
**ADW 4**		**34.18 D**	**32.85 C**	**11.17 D**	**10.03 EF**	**2.757 D**	**2.677 D**	**14.25 C**	**12.67 C**
**TSW 1**		**84.79 A**	**84.47 A**	**25.85 BC**	**25.99 ABC**	**6.210 AB**	**6.273 AB**	**27.16 A**	**27.07 A**
**TSW 2**		**81.76 A**	**85.23 A**	**24.11 C**	**23.56 C**	**5.538 B**	**5.805 B**	**26.21 A**	**25.99 A**
**TSW 3**		**42.86 C**	**34.80 C**	**13.03 D**	**13.65 D**	**2.785 D**	**2.958 D**	**16.55 BC**	**16.53 B**
**TSW 4**		**26.85 E**	**24.48 D**	**10.19 D**	**9.09 F**	**2.337 D**	**2.588 D**	**14.78 C**	**13.94 BC**
	**0mM**	**59.80 B**	**57.59 B**	**17.94 B**	**17.47 B**	**4.259 B**	**4.387 B**	**20.58 B**	**19.77 A**
	**50mM**	**69.03 A**	**68.95 A**	**22.51 A**	**21.93 A**	**5.301 A**	**5.399 A**	**23.97 A**	**14.45 B**
NIW	0mM	84.92 ab	85.22 abc	29.00 a	27.44 ab	5.837 bcd	5.827 c-f	27.97 abc	27.63 ab
	50mM	89.47 a	90.71 a	31.62 a	30.54 a	7.570 a	7.597 a	29.83 a	28.57 ab
ADW 1	0mM	78.03 bc	75.29 d	26.74 ab	25.06 bc	6.140 bc	5.893 cde	26.92 abc	26.34 ab
	50mM	88.60 a	89.61 ab	29.76 a	30.77 a	6.867 ab	7.177 ab	28.34 abc	28.21 ab
ADW 2	0mM	78.46 bc	79.33 cd	22.34 bc	22.38 cd	5.277 cd	5.667 def	24.33 bcd	23.16 bc
	50mM	89.09 a	88.91 ab	29.47 a	28.01 ab	6.803 ab	6.893 abc	27.88 abc	28.31 ab
ADW 3	0mM	50.42 e	44.55 fg	10.57 efg	11.03 fgh	3.503 e	3.610 g	17.84 fgh	13.69 ef
	50mM	59.62 d	61.66 e	15.90 de	14.75 ef	4.797 d	4.810 f	19.93 def	18.02 de
ADW 4	0mM	30.79 fg	27.68 h	10.25 fg	9.12 gh	2.903 e	2.643 gh	12.40 i	10.89 f
	50mM	37.57 f	38.01 g	12.08 efg	10.94 fgh	2.610 e	2.710 gh	16.11 f-i	14.45 def
TSW 1	0mM	80.66 abc	79.19 cd	22.86 bc	22.88 cd	5.517 cd	5.837 c-f	25.09 abc	24.74 ab
	50mM	88.91 a	89.75 ab	28.84 a	29.10 ab	6.903 ab	6.710 a-d	29.24 ab	29.41 a
TSW 2	0mM	75.03 c	81.72 bcd	19.83 cd	18.89 de	4.640 d	5.077 ef	23.45 cde	23.33 bc
	50mM	88.49 a	88.73 ab	28.39 a	28.22 ab	6.437 abc	6.533bcd	28.97 ab	28.65 ab
TSW 3	0mM	36.11 f	23.80 h	11.32 efg	12.92 fg	2.247 e	2.497 h	14.22 ghi	13.68 ef
	50mM	49.61 e	45.80 f	14.73 def	14.38 f	3.323 e	3.420 gh	18.88 efg	19.39 cd
TSW 4	0mM	23.80 g	21.65 h	8.57 g	7.50 h	2.270 e	2.433 h	13.02 hi	14.53 def
	50mM	29.91 fg	27.39 h	11.81efg	10.68 fgh	2.403 e	2.743 gh	16.55 f-i	13.36 ef

*NIW* Nile irrigation water 100%, *ADW1* agricultural drainage water 25%:Nile irrigation water 75%, *ADW2* agricultural drainage water 50%: Nile irrigation water 50%, *ADW3* agricultural drainage water 75%:Nile irrigation water 25%, *ADW4* agricultural drainage water100%, *TSW1* treated sewage water 25%:Nile irrigation water 75%, *TSW2* treated sewage water 50%:Nile irrigation water 50%, *TSW3* treated sewage water 25%: Nile irrigation water 75%, *TSW4* treated sewage water 100%

Values marked with the same letter(s) within the main and interaction impacts are statistically similar using Duncan's multiple range test. Uppercase letter(s) refers to differences within the main effects and lowercase letter(s) refers to differences within the interaction effects

The vegetative growth attributes (plant height; number of leaves on plants; stem diameter/plant, stem, leaf and root fresh weights; root diameter; dry weight of stems, leaves, and roots; leaf area index; and root length) were significantly positively affected by foliar application of 50 mM Glycine betaine (GB) compared with those of unsprayed seedlings (Tables 3, 4 and 5).

**Fig. 1:**
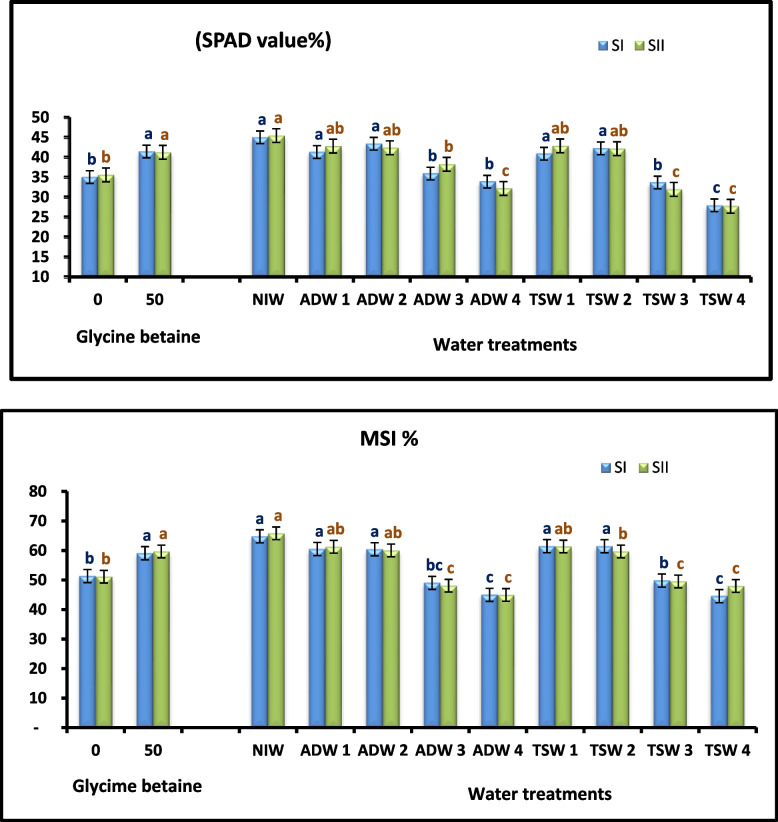
Effect of agricultural drainage water (ADW), treated sewage water (TSW) and glycine betaine (GB) foliar application on relative chlorophyll content (SPAD value %) and membrane stability index (MSI %) of Kapok seedlings during 2021 (SI) and 2022 (SII) seasons

The interaction of Glycine betaine (GB) at a concentration of 50 mM under the water treatments TSW and ADW (Tables 3, 4, and 5) enhanced the abovementioned vegetative growth characteristics (plant height; number of leaves on the plant; stem diameter/plant; fresh weight of the stem; leaves and roots; root diameter; dry weight of the stem, leaves and roots; leaf area index; and root length) of the Kapok plants under irrigation TSW and ADW NIW in both seasons of study (Fig. 1).

#### Physiological parameters

**Fig. 2:**
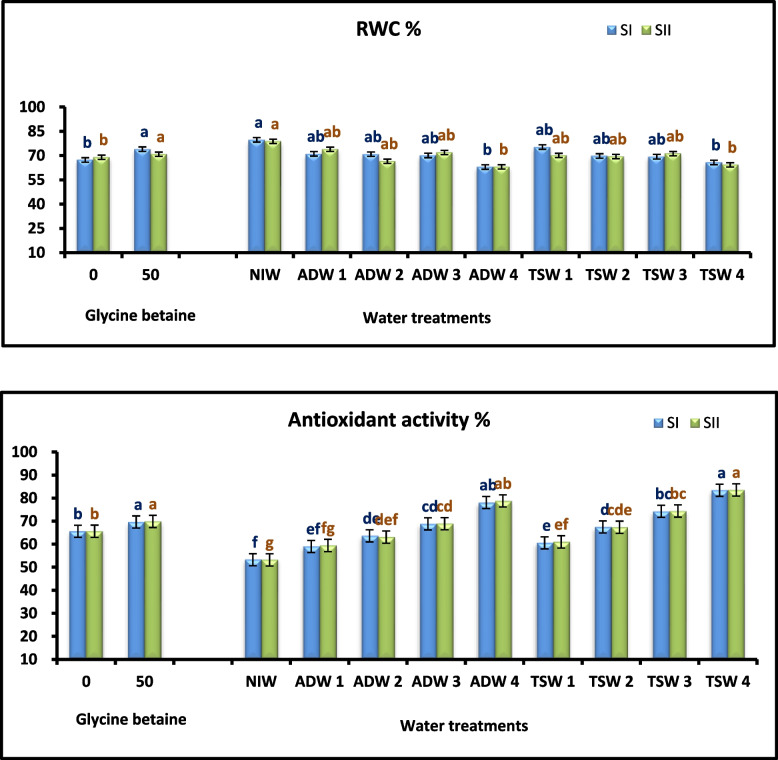
Effect of agricultural drainage water (ADW), treated sewage water (TSW) and glycine betaine (GB) foliar application on relative water content (RWC %) and antioxidant activity % of Kapok seedlings during 2021 (SI) and 2022 (SII) seasons

A negative relationship was found between the physiological parameters and water treatments (Table 6 and Figs. 1, 2). Furthermore, the MSI, RWC, and SPAD values were significantly decreased by irrigation with TSW or ADW at 100%, however, irrigation of Kapok seedlings with TSW or ADW combined with NIW at 25 and 50% did not negatively on affect physiological parameters.

**Table 6:** Main and interaction effects of water treatments (WT) and glycine betaine (GB) foliar application on relative chlorophyll content (SPAD value %), membrane stability index (MSI %),relative water content (RWC %) and antioxidant activity % of Kapok seedlings during 2021 (SI) and 2022 (SII) seasons

Water treatments (WT)	Glycine betaine (GB)	Relative chlorophyll content (SPAD value %)		Membrane stability index (MSI %)		Relative water content (RWC %)		Antioxidant activity (%)	
		**SI**	**SII**	**SI**	**SII**	**SI**	**SII**	**SI**	**SII**
**NIW**		**45.00 A**	**45.43 A**	**64.78 A**	**65.81 A**	**79.68 A**	**78.77 A**	**53.24 F**	**53.17 G**
**ADW 1**		**41.30 A**	**42.78 AB**	**60.48 A**	**61.26 AB**	**71.05 AB**	**73.90 AB**	**58.99 EF**	**59.44 FG**
**ADW 2**		**43.38 A**	**42.37 AB**	**60.43 A**	**60.00 AB**	**70.84 AB**	**66.48 AB**	**63.62 DE**	**63.07DEF**
**ADW 3**		**35.90 B**	**38.23 B**	**49.02 BC**	**48.07 C**	**70.05 AB**	**71.89 AB**	**68.82 CD**	**68.87CD**
**ADW 4**		**33.87 B**	**32.17 C**	**44.96 C**	**44.93 C**	**62.93 B**	**63.04 B**	**78.09 AB**	**78.74 AB**
**TSW 1**		**40.87 A**	**42.83 AB**	**61.46 A**	**61.35 AB**	**75.25 AB**	**70.07 AB**	**60.56 E**	**61.00 EF**
**TSW 2**		**42.22 A**	**42.15 AB**	**61.43 A**	**59.68 B**	**69.75 AB**	**69.41 AB**	**67.46 D**	**67.33 CDE**
**TSW 3**		**33.65 B**	**31.93 C**	**49.81 B**	**49.52 C**	**69.30 AB**	**71.19 AB**	**74.26 BC**	**74.39 BC**
**TSW 4**		**27.95 C**	**27.70 C**	**44.52 C**	**47.97 C**	**65.75 B**	**64.25 B**	**83.39 A**	**83.536 A**
	**0mM**	**35.03 B**	**35.57 B**	**51.34 B**	**51.12 B**	**67.30 B**	**68.95 B**	**65.59 B**	**65.597 B**
	**50mM**	**41.44 A**	**41.23 A**	**59.08A**	**59.68 A**	**73.95 A**	**70.82 A**	**69.62 A**	**69.860 A**
NIW	0mM	40.70 bcd	42.33 abc	62.03abc	63.19 abc	77.10 ab	73.49 ab	51.70 i	55.71 gh
	50mM	49.30 a	48.53 a	67.54 a	70.43 a	91.40 a	78.81 a	54.79 hi	50.63 h
ADW 1	0mM	35.67 d-g	39.07 bcd	53.54 de	55.15 cd	73.65 ab	71.50 ab	57.21 ghi	57.80 fgh
	50mM	46.93 ab	46.50 a	67.43 a	67.37 ab	78.21 ab	78.73 a	60.77 fgh	61.08 d-h
ADW 2	0mM	40.30 bcd	39.33 bcd	56.72 cd	55.30 cd	63.89 b	73.50 ab	62.97 fgh	44.50 e
	50mM	46.47 ab	45.40 ab	64.15 ab	64.69 ab	75.89 ab	76.33 ab	64.27 fgh	62.08 d-h
ADW 3	0mM	34.63 d-g	37.37 cd	44.79 g	45.07 fg	63.00 b	71.46 ab	66.49 efg	67.80 c-f
	50mM	37.17 c-g	39.10 bcd	53.25 def	51.06 d-g	63.62 b	73.63 ab	71.16 b-f	69.94 b-e
ADW 4	0mM	31.50 e–h	29.93 ef	43.17 g	43.55 g	59.11 b	59.46 b	76.37a-e	80.34 ab
	50mM	36.23 d-g	34.40 de	46.75 eg	46.30 efg	68.04 ab	66.51 ab	79.80 abc	77.15 abc
TSW 1	0mM	37.77 c-f	38.83 bcd	54.49 d	54.37 cde	74.70 ab	67.17 ab	58.13 ghi	58.50 e–h
	50mM	43.97 abc	46.83 a	68.42 a	68.34 ab	80.66 ab	71.64 ab	63.00 fgh	63.50 d-g
TSW 2	0mM	38.10 cde	37.77 cd	57.36 bcd	52.70 c-f	71.08 ab	65.00 ab	65.71 fg	65.41 d-g
	50mM	46.33 ab	46.53 a	65.50 a	66.66 ab	78.70 ab	70.28 ab	69.20 def	69.26 b-f
TSW 3	0mM	30.60 fgh	29.17 ef	46.18 g	45.97 efg	61.56 b	65.55 ab	70.75 c-f	70.82 abc
	50mM	36.70 c-g	34.70 de	53.44 de	53.07 c-f	64.30 b	70.89 ab	77.78 a-d	46.92 a-d
TSW 4	0mM	26.03 h	26.30 f	43.82 g	46.73 d-g	62.41 b	60.09 b	80.96 ab	80.19 ab
	50mM	29.87 gh	29.10 ef	45.22 g	49.20 d-g	63.90 b	59.94 b	85.82 a	86.88 a

*NIW* Nile irrigation water 100%, *ADW1* agricultural drainage water 25%:Nile irrigation water 75%, *ADW2* agricultural drainage water 50%: Nile irrigation water 50%, *ADW3* agricultural drainage water 75%:Nile irrigation water 25%, *ADW4* agricultural drainage water100%, *TSW1* treated sewage water 25%:Nile irrigation water 75%, *TSW2* treated sewage water 50%:Nile irrigation water 50%, *TSW3* treated sewage water 25%: Nile irrigation water 75%, *TSW4* treated sewage water 100%

Values marked with the same letter(s) within the main and interaction impacts are statistically similar using Duncan's multiple range test. Uppercase letter(s) refers to differences within the main effects and lowercase letter(s) refers to differences within the interaction effects

The results collectively revealed that 50 mM Glycine betaine (GB) had a positive effect on Physiological parameters (MSI, RWC, and SPAD value) compared with those of seedlings not sprayed with Glycine betaine (GB) (Table 6 and Fig. 2).

Compared with not sprayed with Glycine betaine (GB), the combinations of water treatments and Glycine betaine (GB) (Table 6 and Fig. 2); and Glycine betaine (GB) at a concentration of 50 mM increased the MSI, RWC, and SPAD value, respectively, of seedlings irrigated with 100% TSW and ADW or mixed with NIW.

### Leaf photosynthetic pigment contents

Leaf pigment analysis revealed that, compared with the control, TSW or ADW at 100% (Table 7) significantly, decreased the chlorophyll a, b, and carotenoid contents in both seasons. Under irrigation of with 100% TSW or ADW, the contents of chlorophyll a, b, and carotenoids were significantly lower; however, when Kapok seedlings were irrigated with TSW or ADW in combination with NIW at 25% and 50%, the differences were not significant compared with those under 100% NIW.

**Table 7:** Main and interaction effects of water treatments (WT) and glycine betaine (GB) foliar application on leaf chlorophyll (a), chlorophyll (b) and carotenoid concentrations (mg/mm^2^ fresh weight) of Kapok seedlings during 2021 (SI) and 2022 (SII) seasons

Water treatments (WT)	Glycine betaine (GB)	chlorophyll a (mg/mm^2^)		chlorophyll b (mg/mm^2^)		Carotenoide (mg/mm^2^)	
		**SI**	**SII**	**SI**	**SII**	**SI**	**SII**
**NIW**		**0.309 A**	**0.304 A**	**0.137 A**	**0.187 A**	**0.110 A**	**0.112 A**
**ADW 1**		**0.287 B**	**0.280 AB**	**0.132 A**	**0.133 ABC**	**0.106 A**	**0.105 B**
**ADW 2**		**0.276 B**	**0.276 AB**	**0.123 A**	**0.163 AB**	**0.102 A**	**0.102 B**
**ADW 3**		**0.233 C**	**0.239 C**	**0.108 A**	**0.107 BC**	**0.088 B**	**0.083 D**
**ADW 4**		**0.217 C**	**0.215 C**	**0.224 A**	**0.107 BC**	**0.079 C**	**0.081 D**
**TSW 1**		**0.292 AB**	**0.285 AB**	**0.138 A**	**0.142 ABC**	**0.107 A**	**0.105 B**
**TSW 2**		**0.273 B**	**0.273 B**	**0.134 A**	**0.134 ABC**	**0.104 A**	**0.101 B**
**TSW 3**		**0.235 C**	**0.238 C**	**0.105 A**	**0.115 BC**	**0.089 B**	**0.092 C**
**TSW 4**		**0.217 C**	**0.216 C**	**0.093 A**	**0.096 C**	**0.085 BC**	**0.083 D**
	**0mM**	**0.243 B**	**0.243 B**	**0.110 A**	**0.125 B**	**0.090 B**	**0.089 B**
	**50mM**	**0.277 A**	**0.275 A**	**0.155 A**	**0.138 A**	**0.104 A**	**0.103 A**
NIW	0mM	0.300 ab	0.293 abc	0.132 b	0.146 abc	0.102 bc	0.103 cd
	50mM	0.317 a	0.315 a	0.142 b	0.227 a	0.119 a	0.121 a
ADW 1	0mM	0.268 cde	0.266 bcd	0.120 b	0.125 bc	0.097 cd	0.095 de
	50mM	0.306 ab	0.295 abc	0.140 b	0.142 bc	0.116 a	0.116 ab
ADW 2	0mM	0.252 def	0.257cde	0.115 b	0.124 bc	0.095 cde	0.090 efg
	50mM	0.299 ab	0.296 abc	0.132 b	0.202 ab	0.110 ab	0.113 ab
ADW 3	0mM	0.221 f-i	0.226 def	0.098 b	0.119 bc	0.082 fgh	0.081 gh
	50mM	0.246 efg	0.252 de	0.118 b	0.120 bc	0.094 c-f	0.086 e–h
ADW 4	0mM	0.204 hi	0.201 f	0.081 b	0.096 c	0.076 h	0.078 h
	50mM	0.229 fgh	0.230 def	0.166 a	0.094 c	0.083 e–h	0.084 fgh
TSW 1	0mM	0.278 bcd	0.264 cd	0.135 b	0.134 bc	0.101 bc	0.095 de
	50mM	0.307 ab	0.306 ab	0.141 b	0.150 abc	0.114 a	0.114 ab
TSW 2	0mM	0.248 d-g	0.253 de	0.131 b	0.127 bc	0.095 cde	0.091 ef
	50mM	0.297 abc	0.294 abc	0.138 b	0.141 bc	0.114 a	0.111 bc
TSW 3	0mM	0.219 ghi	0.221 ef	0.098 b	0.108 c	0.085 d-h	0.090 efg
	50mM	0.251 d-g	0.255 cde	0.113 b	0.121 bc	0.094 c-f	0.095 de
TSW 4	0mM	0.194 i	0.200 f	0.079 b	0.089 c	0.080 gh	0.077 h
	50mM	0.240 efg	0.232 def	0.108 b	0.103 c	0.090c-g	0.089 efg

*NIW* Nile irrigation water 100%, *ADW1* agricultural drainage water 25%:Nile irrigation water 75%, *ADW2* agricultural drainage water 50%: Nile irrigation water 50%, *ADW3* agricultural drainage water 75%:Nile irrigation water 25%, *ADW4* agricultural drainage water100%, *TSW1* treated sewage water 25%:Nile irrigation water 75%, *TSW2* treated sewage water 50%:Nile irrigation water 50%, *TSW3* treated sewage water 25%: Nile irrigation water 75%, *TSW4* treated sewage water 100%

Values marked with the same letter(s) within the main and interaction impacts are statistically similar using Duncan's multiple range test. Uppercase letter(s) refers to differences within the main effects and lowercase letter(s) refers to differences within the interaction effects

Foliar application of Glycine betaine (GB) (Table 7), significantly increased the mean values of leaf chlorophyll a, b, and carotenoids. In general, spraying Glycine betaine (GB) at a concentration of 50 mM resulted in significantly greater leaf photosynthetic pigment contents.

The influence of the interaction between water and Glycine betaine (GB) on the leaf photosynthetic pigment content (leaf chlorophyll a, b, and carotenoids) in Table 7 was significant, in both years. The interactive treatment, which induced TSW or ADW at 25 or 50% plus 50 mM Glycine betaine (GB) produced significantly greater amount of the aforementioned leaf photosynthetic pigments.

### Elemental status of the Kapok seedlings

Irrigation with TSW and ADW at 100% significantly decreased the percentages of N, P, K, and Ca, but irrigation with 50% and 25% TSW or ADW in combination with NIW did not significantly affect these elements. Addition, irrigation at 25, 50, and 100% from TSW and ADW significantly increased the percentages of Na, Mn, Pb, Ni, Cu, and Zn, respectively, compared with those of the seedlings irrigated with 100% NIW (Tables 8, 9, and 10).

**Table 8:** Main and interaction effects of water treatments (WT) and glycine betaine (GB) foliar application on Leaf elemental contents (N %, P, K and Ca mg g^−1^) of Kapok seedlings during 2021 (SI) and 2022 (SII) seasons

**Water treatments (WT)**	**Glycine betaine (GB)**	N %		**P mg g-1**		**K mg g-1**		**Ca mg g-1**	
		**SI**	**SII**	**SI**	**SII**	**SI**	**SII**	**SI**	**SII**
**NIW**		**2.783 AB**	**2.749 A**	**0.224 A**	**0.224 A**	**2.63 A**	**2.66 A**	**8.113 A**	**8.22 A**
**ADW 1**		**2.742 AB**	**2.671 A**	**0.217 AB**	**0.215 A**	**2.49 C**	**2.52 B**	**7.838 A**	**7.95 AB**
**ADW 2**		**2.670 C**	**2.639 A**	**0.210 B**	**0.212 A**	**2.51 BC**	**2.50 B**	**7.762 A**	**7.83 AB**
**ADW 3**		**2.282 D**	**2.292 B**	**0.182 C**	**0.167 B**	**2.38 D**	**2.39 C**	**6.460 B**	**6.48 C**
**ADW 4**		**2.234 D**	**2.224 B**	**0.161 D**	**0.152 C**	**2.30 D**	**2.28 DE**	**5.530 C**	**5.73 B**
**TSW 1**		**2.835 A**	**2.744 A**	**0.218 AB**	**0.216 A**	**2.58 AB**	**2.55 AB**	**7.990 A**	**8.17 AB**
**TSW 2**		**2.783 AB**	**2.694 A**	**0.212 B**	**0.214 A**	**2.52 BC**	**2.52 B**	**8.053 A**	**8.13 AB**
**TSW 3**		**2.255 D**	**2.240 B**	**0.179 C**	**0.172 B**	**2.34 D**	**2.34 CD**	**6.607 B**	**6.60 D**
**TSW 4**		**1.966 E**	**2.069 C**	**0.164 D**	**0.164 BC**	**2.31 D**	**2.26 E**	**5.612 C**	**5.61 D**
	**0 mM**	**2.401 B**	**2.402 B**	**0.192 B**	**0.188 B**	**2.38 B**	**2.38 B**	**6.690 B**	**6.73 B**
	**50 mM**	**2.582 A**	**2.558 A**	**0.200 A**	**0.198 A**	**2.52 A**	**2.52 A**	**7.519 A**	**7.59 A**
NIW	0 mM	2.655 bc	2.647 a-d	0.217 b	0.220 a	2.56 b	2.64 ab	7.723 bc	7.94 bc
	50 mM	2.911 a	2.852 a	0.231 a	0.228 a	2.69 a	2.69 a	8.503 a	8.49 ab
ADW 1	0 mM	2.551 c	2.586 cd	0.217 b	0.214 a	2.44 cde	2.46 cd	7.517 bc	7.51 cd
	50 mM	2.833 a	2.757 a-d	0.224 a	0.217 a	2.54 bc	2.58 b	8.160 ab	8.38 ab
ADW 2	0 mM	2.561 c	2.625 bcd	0.206 b	0.208 a	2.42 de	2.42 cd	7.117 cde	7.13 de
	50 mM	2.779 ab	2.653 a-d	0.214 b	0.217 a	2.59 ab	2.61 ab	8.407 a	8.32 ab
ADW 3	0 mM	2.233 de	2.207 ef	0.181 c	0.155 c	2.35 ef	2.35 de	6.280 fg	6.14 g
	50 mM	2.331 d	2.377 e	0.183 c	0.180 b	2.40 e	2.43 cd	6.640 efg	6.82 ef
ADW 4	0 mM	2.217 de	2.217 ef	0.155 e	0.154 c	2.24 g	2.21 gh	5.063 h	5.11 h
	50 mM	2.251 de	2.232 ef	0.167 de	0.150 c	2.37 e	2.35 de	5.997 g	6.04 g
TSW 1	0 mM	2.791 ab	2.674 a-d	0.217 de	0.213 a	2.53 bcd	2.48 c	7.527 bc	7.62 cd
	50 mM	2.879 a	2.813 ab	0.223 a	0.220 a	2.64 ab	2.63 ab	8.453 a	8.71 a
TSW 2	0 mM	2.611 c	2.581 d	0.208 b	0.209 a	2.44 cde	2.45 cd	7.470 bcd	7.55 cd
	50 mM	2.802 ab	2.807 abc	0.217 b	0.218 a	2.60 ab	2.59 ab	8.637 a	8.71 a
TSW 3	0 mM	2.195 de	2.148 f	0.170 d	0.158 c	2.26 fg	2.26 eg	6.393 fg	6.35 fg
	50 mM	2.314 d	2.331 ef	0.189 c	0.187 b	2.41 e	2.43 cd	6.820 def	6.84 ef
TSW 4	0 mM	1.797 f	1.936 g	0.161 de	0.159 c	2.20 g	2.13 h	5.170 h	5.20 h
	50 mM	2.136 e	2.202 ef	0.167 de	0.168 bc	2.42 de	2.39 cd	6.053 g	6.02 g

*NIW* Nile irrigation water 100%, *ADW1* agricultural drainage water 25%:Nile irrigation water 75%, *ADW2* agricultural drainage water 50%: Nile irrigation water 50%, *ADW3* agricultural drainage water 75%:Nile irrigation water 25%, *ADW4* agricultural drainage water100%, *TSW1* treated sewage water 25%:Nile irrigation water 75%, *TSW2* treated sewage water 50%:Nile irrigation water 50%, *TSW3* treated sewage water 25%: Nile irrigation water 75%, *TSW4* treated sewage water 100%

Values marked with the same letter(s) within the main and interaction impacts are statistically similar using Duncan's multiple range test. Uppercase letter(s) refers to differences within the main effects and lowercase letter(s) refers to differences within the interaction effects

**Table 9:** Main and interaction effects of water treatments (WT) and glycine betaine (GB) foliar application on Leaf elemental contents (Na mg g^−1^, Pb, Mn mg/100 g and Cu ppm) of Kapok seedlings during 2021 (SI) and 2022 (SII) seasons

Water treatments (WT)	Glycine betaine (GB)	Na mg g-1		Pb mg/100 g		Cu ppm		Mn mg/100 g	
		**SI**	**SII**	**SI**	**SII**	**SI**	**SII**	**SI**	**SII**
**NIW**		**2.39 E**	**2.37 D**	**41.98 F**	**41.82 F**	**7.91 F**	**8.05 G**	**0.1155 E**	**0.115 D**
**ADW 1**		**2.48 D**	**2.52 C**	**43.96 E**	**44.00 E**	**11.10 E**	**10.93 F**	**0.144 D**	**0.143 C**
**ADW 2**		**2.52 D**	**2.51 C**	**45.35 D**	**45.01 DE**	**16.48 D**	**16.60 D**	**0.207 C**	**0.210 B**
**ADW 3**		**2.74 C**	**2.76 B**	**45.86 CD**	**45.79 CD**	**16.58 D**	**16.64 D**	**0.224 C**	**0.225 B**
**ADW 4**		**2.84 AB**	**2.83 AB**	**46.79 BC**	**46.80 BC**	**19.10 C**	**18.94 C**	**0.256 B**	**0.257 A**
**TSW 1**		**2.47 D**	**2.50 C**	**45.76 CD**	**45.8 BCD**	**12.53 E**	**12.56 E**	**0.132 DE**	**0.133 CD**
**TSW 2**		**2.53 D**	**2.53 C**	**46.73 BC**	**46.87 BC**	**16.54 D**	**16.39 D**	**0.210 C**	**0.208 B**
**TSW 3**		**2.77 BC**	**2.76 B**	**47.22 B**	**47.24 AB**	**24.46 B**	**24.64 B**	**0.255 B**	**0.264 A**
**TSW 4**		**2.90 A**	**2.93 A**	**48.32 A**	**48.38 A**	**33.34 A**	**32.42 A**	**0.288 A**	**0.280 A**
	**0 mM**	**2.71 A**	**2.71 A**	**46.25 A**	**45.21 A**	**18.83 A**	**18.42 A**	**0.2152 A**	**0.217 A**
	**50 mM**	**2.55 B**	**2.56 B**	**44.29 B**	**43.30 B**	**16.30 B**	**16.51 B**	**0.1914 B**	**0.191 B**
NIW	0 mM	2.46 fg	2.44 gh	42.31 f	42.04 f	7.89 h	7.93 h	01160 g	0.115 i
	50 mM	2.31 h	2.31 h	41.65 f	41.59 f	7.93 h	8.17 h	0.1150 g	0.115 i
ADW 1	0 mM	2.55 def	2.57 d-g	45.31 cde	45.58 cde	11.32 fg	10.99 g	0.1430 fg	0.142 hi
	50 mM	2.41 gh	2.42 gh	42.62 f	42.43 f	10.88 g	10.88 g	0.1430 fg	0.145 hi
ADW 2	0 mM	2.59 de	2.59 def	45.10 de	44.50 e	16.99 d	17.03 de	0.2273 cd	0.234 de
	50 mM	2.49 gh	2.42 gh	45.60 cde	45.52 cde	15.97 de	15.98 e	0.1867 e	0.186 fg
ADW 3	0 mM	2.84 bc	2.89 ab	46.58 bcd	46.39 b-e	17.03 d	17.03 de	0.2093 de	0.212 ef
	50 mM	2.62 de	2.62 de	45.13 de	45.18 de	16.13 de	16.26 de	0.2393 bcd	0.239 cde
ADW 4	0 mM	2.87 abc	2.89 ab	47.32 ab	47.32 abc	19.90 c	19.90 c	0.2753 b	0.279 ab
	50 mM	2.91 ab	2.78 bc	46.26 b-e	46.27 b-e	18.30 cd	17.97 d	0.2360 cd	0.236 de
TSW 1	0 mM	2.50 efg	2.51 d-g	46.66 bcd	46.94 a-d	13.72 ef	13.79 f	0.1483 fg	0.150 ghi
	50 mM	2.43 fg	2.49 efg	44.87 e	44.83 de	11.34 fg	11.33 g	0.1153 g	0.115 i
TSW 2	0 mM	2.60 de	2.59 def	46.61 bcd	46.84 a-d	16.73 d	16.50 de	0.2420 bcd	0.239 cde
	50 mM	2.46 fg	2.46 fg	46.84 bc	46.90 a-d	16.34 de	16.28 de	0.1770 ef	0.177 fgh
TSW 3	0 mM	2.90 ab	2.87 ab	47.56 ab	47.55 abc	29.02 b	29.01 b	0.2590 bc	0.277 abc
	50 mM	2.64 d	2.66 cd	46.88 bc	46.92 a-d	19.90 c	20.24 c	0.2517 bc	0.251 bcd
TSW 4	0 mM	2.98 a	3.00 a	48.83 a	48.76 a	36.90 a	37.00 a	0.3163 a	0.304 a
	50 mM	2.82 bc	2.86 b	47.81 ab	48.01 ab	29.95 b	29.89 b	0.2587 bc	0.255 bcd

*NIW* Nile irrigation water 100%, *ADW1* agricultural drainage water 25%:Nile irrigation water 75%, *ADW2* agricultural drainage water 50%: Nile irrigation water 50%, *ADW3* agricultural drainage water 75%:Nile irrigation water 25%, *ADW4* agricultural drainage water100%, *TSW1* treated sewage water 25%:Nile irrigation water 75%, *TSW2* treated sewage water 50%:Nile irrigation water 50%, *TSW3* treated sewage water 25%: Nile irrigation water 75%, *TSW4* treated sewage water 100%

Values marked with the same letter(s) within the main and interaction impacts are statistically similar using Duncan's multiple range test. Uppercase letter(s) refers to differences within the main effects and lowercase letter(s) refers to differences within the interaction effects

**Table 10:** Main and interaction effects of water treatments (WT) and glycine betaine (GB) foliar application on Leaf elemental contents (Zn mg/100 g, Ni ppm and Proline, phenolic content mg g^−1^) of Kapok seedlings during 2021 (SI) and 2022 (SII) seasons

Water treatments (WT)	Glycine betaine (GB)	Zn mg/100 g		Ni ppm		Proline content (mg g^−1^)		Phenolic (mg g^−1^)	
		**SI**	**SII**	**SI**	**SII**	**SI**	**SII**	**SI**	**SII**
**NIW**		**0.210 D**	**0.211 D**	**56.15 G**	**55.85 E**	**0.553 C**	**0.721 BC**	**0.463 E**	**0.497 D**
**ADW 1**		**0.219 DE**	**0.238 BC**	**67.45 F**	**67.53 D**	**0.611 BC**	**0.618 D**	**0.519 DE**	**0.541 CD**
**ADW 2**		**0.232 C**	**0.237 BC**	**72.31 DE**	**71.77 D**	**0.752 A**	**0.742 BC**	**0.549 D**	**0.577 C**
**ADW 3**		**0.241 C**	**0.242 B**	**68.72 EF**	**68.74 D**	**0.792 A**	**0.798 AB**	**0.654 C**	**0.686 B**
**ADW 4**		**0.250 B**	**0.249 B**	**76.79 CD**	**76.80 C**	**0.811 A**	**0.819 A**	**0.759 A**	**0.787 A**
**TSW 1**		**0.219 DE**	**0.229 CD**	**76.39 CD**	**75.86 C**	**0.575 C**	**0.579 DE**	**0.505 DE**	**0.511 D**
**TSW 2**		**0.238 C**	**0.244 B**	**79.50 C**	**79.30 C**	**0.665 B**	**0.708 C**	**0.570 D**	**0.581 C**
**TSW 3**		**0.251 B**	**0.252 AB**	**87.53 B**	**87.81 B**	**0.765 A**	**0.76 ABC**	**0.680 BC**	**0.720 B**
**TSW 4**		**0.269 A**	**0.269 A**	**94.69 A**	**94.68 A**	**0.811 A**	**0.821 A**	**0.724 AB**	**0.732 AB**
	**0 mM**	**0.240 A**	**0.245 A**	**75.54 A**	**77.34 A**	**0.702 B**	**0.704 B**	**0.510 B**	**0.531 B**
	**50 mM**	**0.233 B**	**0.236 B**	**73.47 B**	**72.41 B**	**0.728 A**	**0.732A**	**0.695 A**	**0.720 A**
NIW	0 mM	0.212 h	0.215 de	57.61 h	57.00 f	0.534 g	0.517 g	0.372 h	0.399 g
	50 mM	0.209 h	0.207 e	54.68 h	54.70 f	0.572 fg	0.566 fg	0.555 cde	0.594 cde
ADW 1	0 mM	0.220 fgh	0.256 ab	69.33 fg	69.53 de	0.619 efg	0.616 ef	0.436 fgh	0.467 fg
	50 mM	0.219 gh	0.219 de	65.56 g	65.56 e	0.602 fg	0.619 ef	0.602 cd	0.615 cd
ADW 2	0 mM	0.232 ef	0.234 b-e	76.23 de	75.63 cd	0.760 a-d	0.750 a-d	0.479 efg	0.499 ef
	50 mM	0.232 ef	0.240 bcd	68.39 fg	67.90 e	0.743 a-d	0.735 bcd	0.619 c	0.655 c
ADW 3	0 mM	0.240 e	0.243 bcd	69.64 efg	69.64 de	0.795 ab	0.807 abc	0.553 cde	0.598 cd
	50 mM	0.241 de	0.240 bcd	67.80 fg	67.84 e	0.789 abc	0.788 abc	0.756 b	0.774 b
ADW 4	0 mM	0.254 bcd	0.255 ab	79.11 d	79.11 c	0.811 ab	0.822 ab	0.630 c	0.655 c
	50 mM	0.245 b-e	0.243 bcd	74.47 def	74.49 cd	0.8102 ab	0.816 abc	0.888 a	0.918 a
TSW 1	0 mM	0.226 fg	0.222 cde	78.48 d	77.45 c	0.572 fg	0.583 efg	0.420 gh	0.433 fg
	50 mM	0.211 h	0.218 de	74.31 def	74.27 cd	0.578 fg	0.575 efg	0.589 cd	0.588 cde
TSW 2	0 mM	0.243 cde	0.243 bcd	80.78 cd	80.76 c	0.653 def	0.750 a-d	0.515 def	0.520 def
	50 mM	0.233 ef	0.244 bcd	78.23 d	77.85 c	0.677 c-f	0.666 de	0.625 c	0.642 c
TSW 3	0 mM	0.255 bc	0.253 abc	88.33 b	88.57 b	0.729 b-e	0.7220 cd	0.577 cd	0.600 cd
	50 mM	0.246 b-e	0.252 abc	86.73 bc	87.06 b	0.802 ab	0.808 abc	0.783 b	0.839 ab
TSW 4	0 mM	0.279 a	0.279 a	98.33 a	98.34 a	0.848 a	0.843 a	0.608 cd	0.606 cd
	50 mM	0.258 b	0.259 ab	91.06 b	91.02 b	0.801 ab	0.799 abc	0.839 ab	0.859 ab

*NIW* Nile irrigation water 100%, *ADW1* agricultural drainage water 25%:Nile irrigation water 75%, *ADW2* agricultural drainage water 50%: Nile irrigation water 50%, *ADW3* agricultural drainage water 75%:Nile irrigation water 25%, *ADW4* agricultural drainage water100%, *TSW1* treated sewage water 25%:Nile irrigation water 75%, *TSW2* treated sewage water 50%:Nile irrigation water 50%, *TSW3* treated sewage water 25%: Nile irrigation water 75%, *TSW4* treated sewage water 100%

Values marked with the same letter(s) within the main and interaction impacts are statistically similar using Duncan's multiple range test. Uppercase letter(s) refers to differences within the main effects and lowercase letter(s) refers to differences within the interaction effects

Foliar application of Glycine betaine (GB) 50 mM Kapok to seedlings had positive effects on all the elements, i.e., increasing the N, P, K, and Ca percentages compared with those in seedlings sprayed with water (Tables 8, 9, and 10). In addition, it decreased the concentrations of heavy metals (Cu, Ni, Pb, Zn, Mn, and Na) in Kapok leaves.

The interaction effect between water treatment and foliar application of Glycine betaine (GB) was significant (Tables 8, 9, and 10). It can be inferred that spraying Kapok seedlings with 50 mM Glycine betaine (GB) via irrigation with TSW or ADW at 25 or 50% increase the percentages of N, P, K, and Ca but decreased the concentrations of heavy metals in Kapok leaves (Cu, Ni, Pb, Zn, Mn, and Na).

### Leaf osmoprotectant compounds and antioxidant activity

**Fig. 3:**
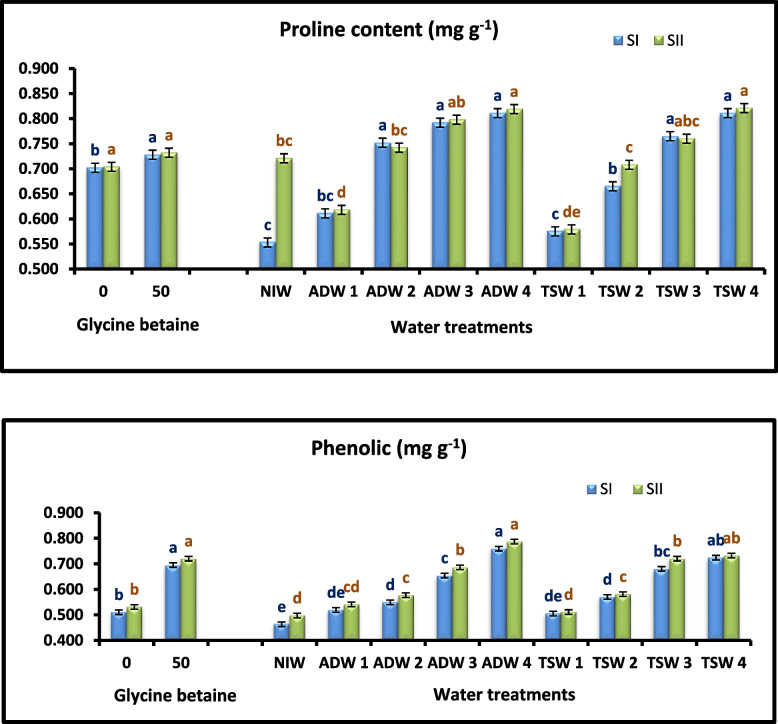
Effect of agricultural drainage water (ADW), treated sewage water (TSW) and glycine betaine (GB) foliar application on Proline and phenolic content of Kapok seedlings during 2021 (SI) and 2022 (SII) seasons

By irrigation with TSW and ADW, the highest contents of total phenolic, proline, and antioxidant activity were obtained. The highest content was produced by irrigation at 100% TSW or ADW (Tables 6 and 10) and (Figs. 2 and 3).

Foliar application of Glycine betaine (GB) caused an increase in the total phenolic content, proline content, and antioxidant activity compared with those of seedlings without foliar Glycine betaine (GB) (Tables 6 and 10) and (Figs. 2 and 3).

The effects of water treatment and Glycine betaine (GB) addition on increasing the antioxidant activity content of dry Kapok leaves were observed (Tables 6 and 10) and (Figs. 2 and 3). The highest total phenolic content, proline content, and antioxidant activity were recorded from plants irrigated with 100% ADW or TSW and those sprayed with 50 mM Glycine betaine (GB) in the first and second seasons, respectively, with significant differences in both seasons.

### Leaf anatomical study

**Fig. 4:**
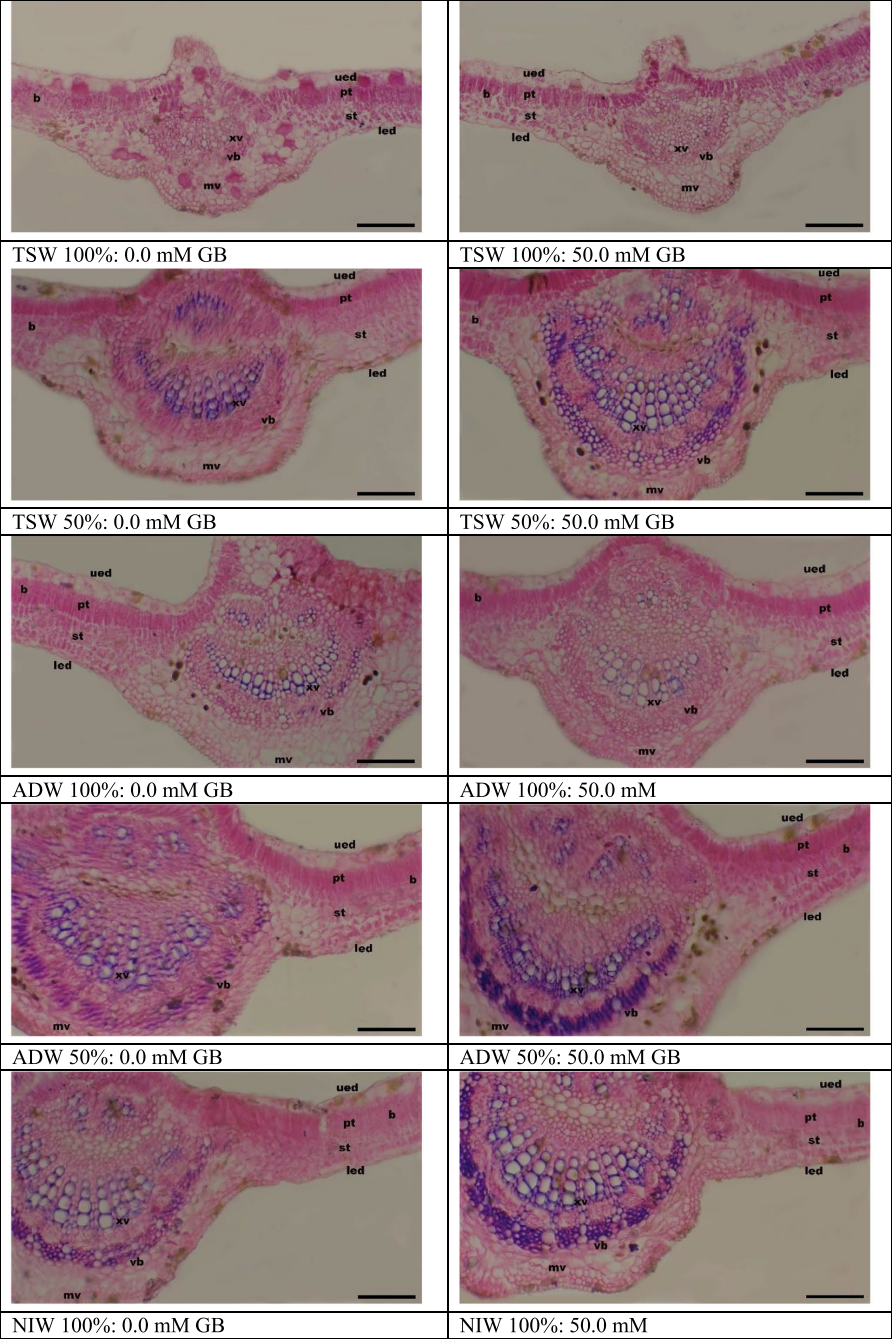
Transverse sections in Kapok leaves treated with treated sewage water (tsw) and agricultural drainage water (ADW) as well as glycine betaine (GB). vb: vascular bundle, mv: mid vein, b: blade, and xv: xylem vessel, ued: upper epidermis, pt: palisade tissue, st: sponge tissue, led: lower epidermis. Bares represent 200 µm

**Fig. 5:**
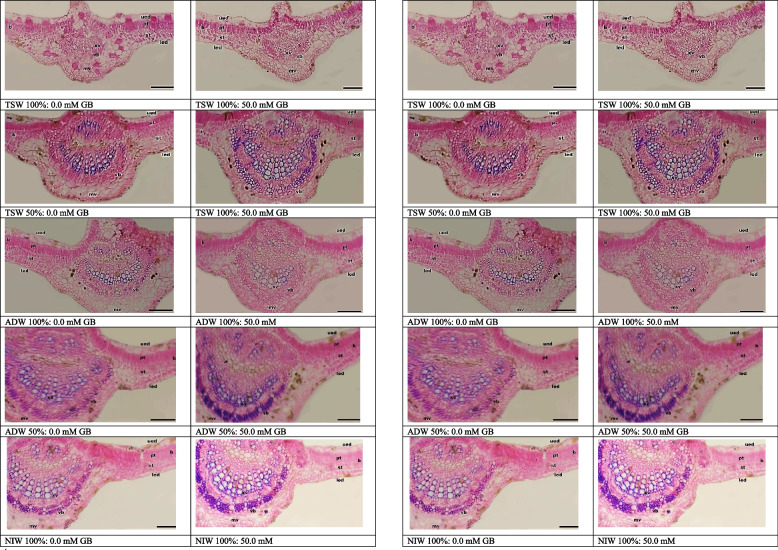
(SII) Transverse sections in Kapok leaves irrigated with treated sewage water (TSW) and agricultural drainage water (ADW) as well as glycine betaine (GB). vb: vascular bundle, mv: mid vein, b: blade, and xv: xylem vessel, ued: upper epidermis, pt: palisade tissue, st: sponge tissue, led: lower epidermis. Bares represent 200 µm. (SII) Transverse sections in Kapok leaves irrigated with treated sewage water (TSW) and agricultural drainage water (ADW) as well as glycine betaine (GB). vb: vascular bundle, mv: mid vein, b: blade, and xv: xylem vessel, ued: upper epidermis, pt: palisade tissue, st: sponge tissue, led: lower epidermis. Bares represent 200 µm

The data in Tables 11 and 12 and Figs. 4 and 5 show comparisons between seedlings irrigated with NIW in terms of leaf thickness and leaf mid-vein, which respond differently to TSW and ADW irrigation. Compared with NIW, Irrigation with TSW or ADW at 100% caused a decrease in almost all the anatomical features of the Kapok plant leaves including the height of the mid-vein and height, width of the vascular bundle and diameter of the xylem vessel, leaf blade thickness, palisade tissue thickness, spongy tissue thickness and upper epidermis layer NIW. In addition, irrigation with TSW and ADW at 25% and 50% had no significant differences from irrigating with NIW.

**Table 11:** Main and interaction effects of water treatments (WT) and glycine betaine (GB) foliar application on anatomical structures of mid-vein (average height of mid-vein, average height of vascular bundle, average width of vascular bundle and average diameter of xylem vessel µm) of Kapok seedlings during 2021 (SI) and 2022 (SII) seasons

Water treatments (WT)	Glycine betaine (GB)	average height of Mid-vein (µm)		Average height of vascular bundle (µm)		Average width of vascular bundle (µm)		Average diameter of xylem vessel (µm)	
		**SI**	**SII**	**SI**	**SII**	**SI**	**SII**	**SI**	**SII**
**NIW**		**1278.82 A**	**1257.69 A**	**359.72 A**	**360.32 A**	**853.02 A**	**850.78 A**	**43.667 A**	**42.83 A**
**ADW 1**		**1232.47 A**	**1252.66 A**	**358.89 A**	**359.44 A**	**796.42 AB**	**798.42 AB**	**42.167 A**	**41.50 A**
**ADW 2**		**1132.69 AB**	**1150.19 AB**	**338.77 A**	**339.32 A**	**762.25 BC**	**765.91 BC**	**41.333 A**	**41.17 AB**
**ADW 3**		**971.77 D**	**1011.97 D**	**266.29 B**	**264.56 B**	**632.61 D**	**631.89 D**	**28.500 CD**	**28.67 CD**
**ADW 4**		**834.71 E**	**823.67 E**	**193.09 C**	**195.82 C**	**430.12 E**	**430.73 E**	**26.667 D**	**26.50 D**
**TSW 1**		**1151.06 B**	**1146.71 B**	**354.42 A**	**355.43 A**	**793.58 B**	**793.91 B**	**42.500 A**	**41.33 AB**
**TSW 2**		**1055.17 C**	**1048.63 C**	**319.87 A**	**322.28 A**	**735.94 C**	**734.95 C**	**41.000 A**	**40.17 AB**
**TSW 3**		**781.79 E**	**758.98 E**	**246.61 B**	**247.14 B**	**614.03 D**	**610.75 D**	**37.667 B**	**37.67 B**
**TSW 4**		**597.83 F**	**603.99 EF**	**177.49 C**	**169.71 C**	**378.59 F**	**380.21 F**	**30.333 C**	**30.17 C**
	**0mM**	**949.341 B**	**961.450 B**	**269.36 B**	**270.67 B**	**641.09 B**	**641.55 B**	**35.667 B**	**35.444 B**
	**50mM**	**1058.73 A**	**1053.22 A**	**311.79 A**	**310.22 A**	**686.80 A**	**686.78 A**	**38.519 A**	**37.889 A**
NIW	0mM	1299.57 ab	1291.17 ab	339.46 a-d	340.24 abc	850.11 a	854.46 a	42.667 ab	42.00 ab
	50mM	1358.08 a	1324.22 a	379.98 ab	380.52 a	849.93 a	847.11 a	44.667 a	43.67 a
ADW 1	0mM	1190.47 bc	1216.43 b	331.49 a-d	332.63 abc	776.69 bc	775.92 bc	41.000 abc	40.67 ab
	50mM	1274.47 ab	1288.89 ab	386.296 a	386.25 a	820.15 ab	820.91 a	43.333 ab	42.33 ab
ADW 2	0mM	1102.21 cd	1130.85 cd	314.85 a-e	314.13 bcd	740.73 c	743.13 c	41.00 abc	40.67 ab
	50mM	1163.18 c	1169.54 c	362.70 ab	364.49 ab	743.77 c	748.69 c	41.667 abc	41.67 ab
ADW 3	0mM	905.73 fg	951.68 ef	242.98 fgh	244.10 efg	617.52 d	619.18 de	24.667 ef	25.33 d
	50mM	1037.81 de	1032.26 de	289.61 c-f	284.83 cde	647.69 d	644.61 d	32.333 d	32.00 c
ADW 4	0mM	725.08 h	722.03 gh	180.67 hi	186.92 ghi	362.75 g	366.16 h	24.000 f	24.00 d
	50mM	944.32 ef	925.31 ef	205.51 ghi	204.60 f-i	497.48 e	495.29 f	29.333 d	29.00 cd
TSW 1	0mM	1101.14 cd	1095.49 cd	333.21 a-d	337.11 abc	772.24 bc	773.27 bc	37.000 ab	38.67 ab
	50mM	1251.98 abc	1265.92 abc	375.63 ab	373.82 ab	814.91 ab	814.55 ab	43.000 ab	42.00 ab
TSW 2	0mM	1014.19 de	1027.55 de	285.66 def	284.14 cde	731.99 c	729.64 c	40.33 abc	40.00 ab
	50mM	1096.14 cd	1079.72 cd	354.09 abc	360.30 ab	739.88 c	740.26 c	41.667 ab	40.33 ab
TSW 3	0mM	720.40 h	703.63 h	235.16 fgh	236.31 e–h	587.51 d	579.73 e	36.667 c	37.00 b
	50mM	843.18 g	814.32 g	258.07 efg	257.92 def	640.55 d	641.77 d	38.667 bc	38.33 ab
TSW 4	0mM	585.27 i	569.21 i	160.72 i	160.37 i	330.33 g	332.53 h	28.67 de	28.67 cd
	50mM	610.39 i	638.76 hi	194.28 ghi	179.20 hi	426.86 f	427.89 g	32.00 d	31.67 c

*NIW* Nile irrigation water 100%, *ADW1* agricultural drainage water 25%:Nile irrigation water 75%, *ADW2* agricultural drainage water 50%: Nile irrigation water 50%, *ADW3* agricultural drainage water 75%:Nile irrigation water 25%, *ADW4* agricultural drainage water100%, *TSW1* treated sewage water 25%:Nile irrigation water 75%, *TSW2* treated sewage water 50%:Nile irrigation water 50%, *TSW3* treated sewage water 25%: Nile irrigation water 75%, *TSW4* treated sewage water 100%

Values marked with the same letter(s) within the main and interaction impacts are statistically similar using Duncan's multiple range test. Uppercase letter(s) refers to differences within the main effects and lowercase letter(s) refers to differences within the interaction effects

**Table 12:** Main and interaction effects of water treatments (WT) and glycine betaine (GB) foliar application on (average thickness of leaf blade, average the upper epidermis layer, average thickness of palisade tissue and average width of vascular bundle µm) of Kapok seedlings during 2021 (SI) and 2022 (SII) seasons

Water treatments (WT)	Glycine betaine (GB)	Average thickness of leaf blade (µm)		Average the upper epidermis layer (µm)		Average thickness of palisade tissue (µm)		Average thickness of spongy tissue (µm)	
		**SI**	**SII**	**SI**	**SII**	**SI**	**SII**	**SI**	**SII**
**NIW**		**278.79 A**	**281.17 A**	**45.12 A**	**44.72 A**	**82.46 A**	**82.11 A**	**111.80 A**	**110.01 A**
**ADW 1**		**263.93 AB**	**269.38 AB**	**44.14 AB**	**43.00 AB**	**79.87 A**	**79.66 A**	**103.57 AB**	**101.09 AB**
**ADW 2**		**259.82 AB**	**256.93 AB**	**42.28 AB**	**42.68 B**	**76.47 A**	**77.83 AB**	**84.41 AB**	**97.64 AB**
**ADW 3**		**223.98 DE**	**225.15 D**	**39.21 B-E**	**38.87C**	**65.84 B**	**65.24 C**	**74.92 D**	**74.27 D**
**ADW 4**		**207.05 E**	**208.45 E**	**38.29 CD**	**38.05 C**	**60.25 BC**	**60.20 D**	**67.08 DE**	**65.87 EF**
**TSW 1**		**254.31 AB**	**254.20 BC**	**44.59 AB**	**43.86AB**	**80.89 A**	**79.92 A**	**96.93 B**	**97.75 AB**
**TSW 2**		**235.57 CD**	**237.14 D**	**39.68 A-D**	**39.02 C**	**75.15 A**	**74.24 B**	**84.31 C**	**83.17 C**
**TSW 3**		**208.09 E**	**209.19 E**	**33.68 E**	**32.77 D**	**60.73 BC**	**60.08 D**	**70.65 D**	**70.04 DE**
**TSW 4**		**178.75 F**	**179.41 F**	**34.39 DE**	**34.04 D**	**54.76 C**	**54.01 E**	**62.28 E**	**61.90 F**
	**0mM**	**226.555 B**	**227.791 B**	**39.68 A**	**38.943 A**	**69.123 B**	**68.587 B**	**80.595 B**	**79.663 B**
	**50mM**	**237.956 A**	**238.883 A**	**40.625 A**	**40.172 A**	**72.301 A**	**71.703 A**	**85.617 A**	**84.728 A**
NIW	0mM	275.26 ab	279.19 a	38.36 a-d	44.23 a	76.76 a-e	76.57 d	109.60 ab	106.98 ab
	50mM	282.33 a	283.15 a	40.06 a-d	45.21 a	88.16 a	87.65 a	114.00 a	113.05 a
ADW 1	0mM	260.42 a-d	262.07 ab	43.26 ab	41.43 abc	76.87 a-e	77.11 cd	91.86 cd	91.62 cd
	50mM	267.44 abc	266.68 a	45.03 a	44.58 a	82.87 ab	82.20 bc	99.27 bc	98.57 bc
ADW 2	0mM	242.09 a-e	241.98 bcd	42.66 ab	41.37 abc	77.66 abc	76.62 d	80.15 de	79.71 fg
	50mM	237.54 b-f	239.88 bcd	41.89 abc	41.99 abc	75.29 a-f	75.04 d	88.67 cd	87.57 def
ADW 3	0mM	216.60 e–h	217.29 de	38.36 a-d	37.96 c	64.44 d-h	64.21 e	74.71 ef	74.05 gh
	50mM	231.36 c-g	233.02 cd	40.06 a-d	39.78 bc	67.24 c-g	66.28 e	75.13 ef	74.50 gh
ADW 4	0mM	197.49 f-i	198.91 ef	38.87 a-d	38.11 c	58.35 gh	58.25 f	64.11 fg	63.02 ij
	50mM	216.60 e–h	217.99 de	37.70 a-d	37.99 c	62.14 gh	62.16 ef	70.04 efg	68.71 hi
TSW 1	0mM	239.66 b-f	240.65 bcd	42.90 ab	42.55ab	76.90 a-e	75.82 d	89.83 cd	89.09 de
	50mM	268.97 abc	267.76 a	46.27 a	45.17 a	84.87 ab	84.02 ab	104.04 sb	102.42 b
TSW 2	0mM	241.66 a-e	243.19 bc	39.12 a-d	38.95bc	74.33 b-f	73.49 d	81.33 de	80.65 efg
	50mM	229.49 c-g	231.08 cd	40.24 a-d	39.09 bc	75.97 b-f	74.99 d	87.30 cd	85.68 def
TSW 3	0mM	193.60 ghi	194.19 fg	32.65 d	31.67d	59.57 gh	58.57 f	74.29 ef	73.56 gh
	50mM	222.58 d-h	224.19 cd	34.71 bcd	33.87 d	61.88 gh	61.58 ef	67.01 fg	66.52 hig
TSW 4	0mM	172.21 i	172.63 g	34.74 bcd	34.20d	57.23 gh	56.64 f	59.47 g	58.28 j
	50mM	185.29 hi	186.20 fg	34.03 cd	33.87 d	52.29 h	51.38 g	65.08 fg	65.53 hij

*NIW* Nile irrigation water 100%, *ADW1* agricultural drainage water 25%:Nile irrigation water 75%, *ADW2* agricultural drainage water 50%: Nile irrigation water 50%, *ADW3* agricultural drainage water 75%:Nile irrigation water 25%, *ADW4* agricultural drainage water100%, *TSW1* treated sewage water 25%:Nile irrigation water 75%, *TSW2* treated sewage water 50%:Nile irrigation water 50%, *TSW3* treated sewage water 25%: Nile irrigation water 75%, *TSW4* treated sewage water 100%

Values marked with the same letter(s) within the main and interaction impacts are statistically similar using Duncan's multiple range test. Uppercase letter(s) refers to differences within the main effects and lowercase letter(s) refers to differences within the interaction effects

Compared with non-Sprayed seedlings, those sprayed with 50 mM Glycine betaine (GB) presented greater leaf thickness (height of the mid-vein and height, width of the vascular bundle, and diameter of the xylem vessel) and leaf mid-vein thickness (leaf blade thickness, palisade tissue thickness, spongy tissue thickness, and upper epidermis layer) (Tables 11 and 12) (Figs. 4 and 5).

The interaction effect between water treatment and foliar application (Tables 11 and 12 and Figs. 4 and 5) was significant. It can be inferred that spraying Kapok seedlings with Glycine betaine (GB) along with irrigation with TSW and ADW resulted in a greater increase in leaf thickness and leaf mid-vein than did water treatment or Glycine betaine (GB) alone in both seasons.

## Discussion

The use and reuse of treated sewage water and ADW can augment the available water in several countries where fresh water is in short supply. A study conducted [29] on *Populus nigra* and *P. alba* declared that TSW decreases leaf number. Ali et al., [30] On *Swietenia mahagoni* stated that TSW reduced root length. Bañón et al., [31] On polygala and lantana plants discovered that TSW reduces plant dry weight [32] on vegetable crops (tomato, radish and lettuce) proclaimed that TSW decreases all vegetative growth. This study attributed such negative effect to the toxic levels of heavy metals in wastewater. Similar results were obtained in Tables 3, 4, and 5. In contrast, [30] studied *Swietenia mahagoni* seedlings, [33] studied *Tipuana speciosa*, and [34] studied *Myrtus comunis, Eucalyptus camaldulensis,* and* Cupressus sempervirens* and announced that secondary TSW increased vegetative growth. [35] advertised that secondary TSW had no significant effect on vegetative growth in *Calotropis procera* plants. The decreased in vegetative growth with increasing TSW might be associated with heavy metals, [36] presented that heavy metals problems could lead to a decrease in nutrient uptake plants, disorders in nutrient metabolism, and a decrease in nitrogen or phosphorous fixation in plants.

Table 6 confirms the findings [37] for polygala and lantana plants, who recited that, compared with well water, TSW reduced the relative chlorophyll content (SPAD) and that mixed water (TSW with NIW 50:50) led to grater leaf SPAD values than did TSW. The data in Table 7 show that TSW and ADW decreased leaf photosynthesis, These findings were confirmed by [32], who studied three vegetable crop plants (tomato, radish, and lettuce) and recounted that compared with fresh water, TSW decreases chlorophyll a, b, and total carotenoids, possibly because the heavy metal content in the irrigation water increased, which results from the negative effects of the toxic levels of heavy metals in the wastewater. In addition to the effects of TSW on leaf area [37, 38] apprised that increased cadmium levels in water soils might prohibit soil productivity, whereas very low cadmium concentrations could decrease the biological processes in plants, including the process of photosynthesis, and this is true for lead. Additionally, [39] acquainted that, compared with tap water, *Terminalia angustifolia, Rademachera ignea* and *Ficus mango*, TSW decreased the total carotenoid content in leaves. In contrast, [34] adduced that, compared with tap water, Amin (*Myrtus comunis, Eucalyptus camaldulensis*, and *Cupressus sempervirens*) resulted in increased chlorophyll (a) content in leaves.

The results (Tables 9 and 10) revealed that TSW is rich in heavy metals, which is in agreement with the findings of [34] on (*Myrtus comunis, Eucalyptus camaldulensis, and Cupressus sempervirens*), [40] (*Taxodium distichum, Cupressus sempervirens, Khaya senegalensis,* and* Simmondsiaco hinensis*), they who notified that the total Pb, Mn, Cu, Zn, and Ni contents increased in irrigated plants with secondary TSW compared with those in well water. Additionally, [30] studied *Swietenia mahagoni* seedlings, and [41] investigated *Acacia saligna, Acacia stenophylla,* and* Ceratonia siliqua* and informed that, compared with tap water, secondary TSW increased the Pb, Mn, Cu, Zn, and Ni contents in leaf plants. Which could be due to an increase in the occupancy of the root zone caused by the use of TSW which is reflected in root uptake. The data revealed that TSW and ADW affected the element contents of the leaves of the plants. These findings were confirmed by [39] on *Terminalia angustifolia, Rademachera ignea,* and* Ficus mango* who communicated that, compared with well water, TSW decreased the total N and P contents in leaves. Ahmed et al., [42] proclaimed that stress could be indirectly associated with low N concentrations in plants because of the role of chloride ions. In contrast, the results obtained by [41] on *Acacia saligna, Acacia stenophylla,* and* Ceratonia siliqua* indicated that the total N, P, K, Ca, and Na contents increased with secondary TSW compared with those in well water. Ashraf and O'leary [43] stated that the total K^+^ content decreased more with secondary TSW than with tap water. The reduction in the K^+^ concentration in the plants might be attributed to the Na + ions that could partially replace the K^+^ ions in the plant tissue, so the more concentrated the salinity treatment, was the greater the degree of Na + substitution and consequently, the decrease in K + ions in the plant tissue.

It is discovered that the primary producers' (plants') development, metabolism, and photosynthetic activity are all impacted by the intake of lead [44]. According to [45], plants that are exposed to heavy metals experience oxidative stress, which damages their cells. Additionally, plants accumulate metal ions that upset the ionic balance within cells [46]. Pb can cause morphological, physiological, and biochemical issues in plants, but it serves no biological purpose [47].

Numerous enzymes from various metabolic pathways are similarly impacted by lead treatment. One of the key enzymes in the elimination of harmful peroxides is chloramphenicol acetyltransferase (CAT), an oxidoreductase. It breaks down H_2_O_2_ into molecular oxygen and water. At lower lead concentrations, CAT activities in cuttings and seedlings dramatically increased, whereas at higher lead concentrations, they decreased. Lead also inhibits the activities of reductive pentose phosphate pathway enzymes [48].

The growth of α-amino levulinate dehydrogenase, an essential enzyme for chlorophyll production, is severely inhibited by lead ions. In the presence of lead, ATP synthase/ATPase inhibition was found to be high. Because lead is essential for mitochondria, plant cells showed a decrease in respiration rate as lead concentrations increased. At greater lead concentrations, respiratory inhibition is seen. Although it is shown at lower concentrations, a stimulation of respiration is seen in isolated protoplasts, mitochondria, detached leaves, and whole plants. By reducing their photocatalytic activity, Pb (NO_3_)_2_ in *Ceratopyllum dersum* plants alters the structure of chlorophyll [49].

Numerous factors, including the anatomical and physiological characteristics of the tissue constituent and its structure, the physio-chemical properties of metal ions that prevent their transport over the plant, the efficacy of physiological barriers limiting the radial transport of lead, and the classification of a plant as either an excluder or hyper accumulator type, influence how much lead is absorbed by metal-accumulating tissues [50].

When it comes to the simplistic ions, metal-collecting tissues engage in pericyclic functions [51]. In order to accumulate and deliver metals to the circulatory system, this tissue's primary role is to redistribute ions across the central cylinder's perimeter [52]. According to [53], the lead content of different plant organs tends to decrease as follows: roots > leaves > stem > inflorescence > seeds. In order for plants to thrive, photosynthesis is essential. However, lead buildup in plants has other impacts, such as lowering photosynthetic rate, halting chlorophyll synthesis, affecting the Calvin cycle, and causing a CO2 shortage that closes stomata [54].

Changes in lipid composition caused by lead have a greater effect on chlorophyll B than on chlorophyll A. Fruits and flowers grow differently when photosynthesis is disrupted [55].

The results in Table 10 and Fig, 3 indicate that TSW and ADW increased the total phenolic content and proline content, which is in agreement with the finding of [56] on *Origanum syriacum*, *Mentha Spicata*, *Micromeria fruticose*, Rosemary (*Rosmarinus officinalis*), and *Salvia Fruticose*, who declared that TSW increased the total phenolic content in plants more than well water did. In addition [57, 58] and announced that plants can accumulate phenolic compounds that could play an important role in relieving the harm resulting from environmental stresses. However, [59] demonstrated that phenols have antioxidant activity that could scavenge ROS under abiotic stress in *Hypericum pruinatum*, [60] advertised that TSW led to an increase in the content of proline in plants, which was attributed to high levels of heavy metals in sewage water. In addition [61] announced that the high level of proline accumulation in stressed *Crotalaira striata* plants could be an adaptation to compensate for the energy needed for growth and survival and therefore help the plant tolerate stress. Ahmed et al., [42] declared that proline accumulation can be considered a protective adaptation. In addition, plants survival under stress conditions relies on metabolic process regulation and the ratio of the protective intermediate to the toxic intermediate. Aldesuquy [62] stated that proline could be a cytoplasm-protective osmolyte that is vital for adaptation to stress. Additionally, increased proline concentrations can be good indicators of plant stress tolerance. In contrast, [35] studied *Calotropis procera* seedlings communicated that irrigation with TSW did not significantly affect the proline content of leaves.

[63] proclaimed that sewage water at a concentration of 100% caused a significant decrease in leaf thickness and ground tissue thickness. However, at concentrations of 25% and 50%, increased ground leaf thickness and ground tissue thickness were also detected. Additionally, at all the concentrations, the phloem area and number of opened stomata on both the upper and lower epidermis decreased in the flag leaves of the wheat plants. Additionally, [64] informed that water stress resulted in considerable decreases in leaf and ground tissue thickness, metaxylem and xylem vessel area, and vascular bundle areas in wheat plants.

In this study, we found that secondary TSW increased antioxidant activity in Kapok seedlings (Table 6 and Fig. 2). This study is in agreement with [65] on *Origanum vulgare* and [66] on *Origanum syriacum* var. syriacum, who reported that secondary TSW increased antioxidant activity in seedlings. Abd El-Baky et al., [67] notified that there is an antioxidant defense mechanism in plants exposed to stress. This defense mechanism includes enzymatic and nonenzymatic antioxidants. Kähkönen et al., [68] adduced that mechanisms that decrease reactive oxygen species (ROS) and improve the antioxidant enzyme system of plant play necessary roles in increasing plant tolerance under environmental stress conditions. Munné-Bosch and Alegre [69] acquainted that decreasing the ROS levels during oxidative stress conditions motivated the production of antioxid apprised ants, indicating that plants are subjected to less than optimal conditions. Mittler [70] recounted that chloroplasts are protected from oxidative stress through the regulation of oxidation products. Amin and Al-Atrash [71] recited that heavy metals contained in TSW might increase reactive oxygen production and antioxidant activity in plants. In addition, increased antioxidant content and antioxidant activity have been observed in several plants response to environmental stresses.

Foliar application at Glycine betaine (GB) enhances the vegetative growth of Kapok seedlings (Tables 3, 4, and 5). Similar results were obtained by [72] for *Populus nigra* seedlings, [73] for oat (*Avena sativa*), [74] for *Dalbergia odorifera* seedlings, [75] for *Dalbergia odorifera* seedlings. Kanu et al., [76] presented that Glycine betaine (GB) has a defensive effect on peroxidation-linked membrane deterioration and the ability to scavenge free radicals, in perennial ryegrass [77] indicating that Glycine betaine (GB) enhances plant growth under stress conditions, possibly through osmotic protection.

In general, previous studies indicated that Glycine betaine (GB) increased the physiological parameters of plants, [78] attended that Glycine betaine (GB) increased the SPAD value and MSI during drought stress in *Prunus persica,* and [79] indicated that Glycine betaine (GB) increased the MSI and RWC in *Tagetes erecta*. In addition, [80] studied safflower (*Carthamus tinctorius*), [75] on *Dalbergia odorifera seedlings*, and [79] studied *Tagetes erecta* plants and illustrated that Glycine betaine (GB) increased RWC in these plants. These studies confirmed the results in two seasons.

Glycine betaine (GB) application improved the N, P, K, Ca, phenolic and proline contents in Kapok seedlings (Tables 8 and 9), likewise decreasing the Na^+^ content. Shehzadi et al., [72] advertised that Glycine betaine (GB) increased the N, P, and K contents in on *Populus nigra* plants. Sobahan et al., [81] announced that in contrast with untreated plants, Glycine betaine (GB)-treated plants can consume more nutrients, which is reflected in overall plant growth. In addition, [82] declared that Glycine betaine (GB) could play a role in preserving cytosolic K^+^ homeostasis by repressing Na^+^ enhanced apoplectic flow to decrease Na + uptake. Shehzadi et al., [72] Studied *Populus nigra* seedlings, and [76] studied perennial ryegrass and stated that Glycine betaine (GB) decreased the Na^+^ content in leaf plants. Cisse et al., [73] studied the oat *Avena sativa*, [79] studied *Tagetes erecta* plants, and [83] studied *Ocimum basilicum* proclaimed that Glycine betaine (GB) increased the total phenolic in plants. In contrast, [84] communicated that Glycine betaine (GB) decreased the total phenolic content on *Dalbergia odorifera* plants. Shehzadi et al., [72] On *Populus nigra* seedling, [80] studied safflower (*Carthamus tinctorius*) [74] studied *Dalbergia odorifera* seedlings, and [73] investigated *Avena sativa* L. and reported that Glycine betaine (GB) increased the proline content in plants. In contrast, [76] studied *Lolium perenne*, and [75] studied *Dalbergia odorifera*, and illustrated that Glycine betaine (GB) had no significant effect on the proline content of plants.

In previous studies in which Glycine betaine (GB) application improved leaf midrib thickness and leaf thickness, [73] studied oat (*Avena sativa* L.) and [64] studied two wheat cultivars and announced that Glycine betaine (GB) foliar application increased leaf thickness and leaf midrib thickness.

The data in Table 6 and Fig. 2 show that Glycine betaine (GB) enhanced antioxidant activities. This study confirmed the findings of [74] on *Dalbergia odorifera* seedlings and [75] on *Dalbergia odorifera*, who advertised that Glycine betaine (GB) increased antioxidant activity in leaf plants. Farooq et al., [85] declared that under abiotic stresses, Glycine betaine (GB) could defend plant cells against oxidative stress by maintaining the antioxidant system. [86] stated that the exogenous application of Glycine betaine (GB) in several plants relieves stress-negative effects on photosynthesis by scavenging ROS through the activation of antioxidant activities. An increase in antioxidant activities in *Oryza sativa* under stress was also demonstrated.

## Conclusion

Egypt suffers from a severe shortage of fresh water and is considered one of the dry countries and the per capita share of fresh water is low. Egypt is also affected by the phenomenon of climate change like the countries of the world, especially the rise in its average temperature in the summer. There are vast areas of desert lands in Egypt that are not used for agriculture, especially forest trees. From here, the idea of ​​producing Kapok seedlings irrigated with agricultural drainage water and sewage emerged, saving half the amount of fresh irrigation water from the Nile River and improving the efficiency of using irrigation water, because, the results showed that irrigated Kapok seedlings with ADW and TSW were used in in mixing ratios with NIW at 50% give the better results like irrigated with 100% NIW. This is also through the use of antioxidants, glycine betadine, because it is very important in resisting the damage resulting from stress from irrigation with water of different quality. Antioxidants also play a defensive role in plants against various stress factors Therefore, Glycine betaine (GB) may have a protective effect on peroxidation-linked membrane deterioration, scavenge free radicals and provide osmotic protection.

## Supplementary Information

Supplementary Material 1.

Supplementary Material 2.

## Data Availability

"All data generated or analysed during this study are included in this manuscript" “Data is provided within the manuscript or supplementary information files”.
