# Supplementary material for: Response of Kapok seedlings were irrigated with water of different qualities and heavy metal contents for foliar application of antioxidants
Source: BMC Plant Biol. 2025 Jan 3;25:11. doi: 10.1186/s12870-024-05902-y (PMC11697924; doi:10.1186/s12870-024-05902-y)
Supplement: Supplementary file 1 — Supplementary Material 1. [file 12870_2024_5902_MOESM1_ESM.docx]

| 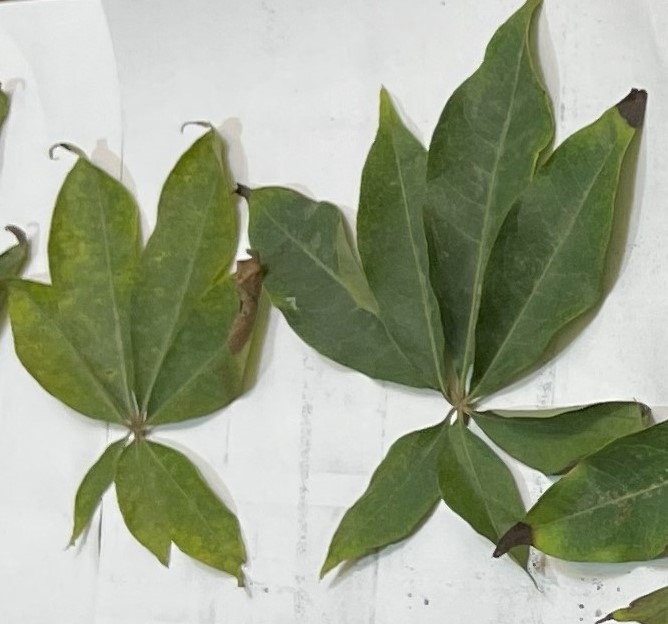 | 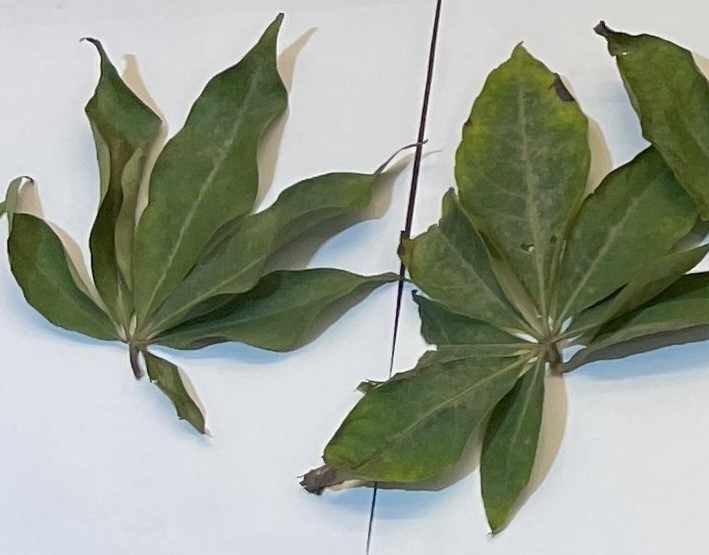 |
| --- | --- |
| TSW 100%: 0.0 mM GB- TSW 100%: 50.0 mM GB | ADW 100%: 0.0 mM GB- ADW 100%: 50.0 mM GB |
| 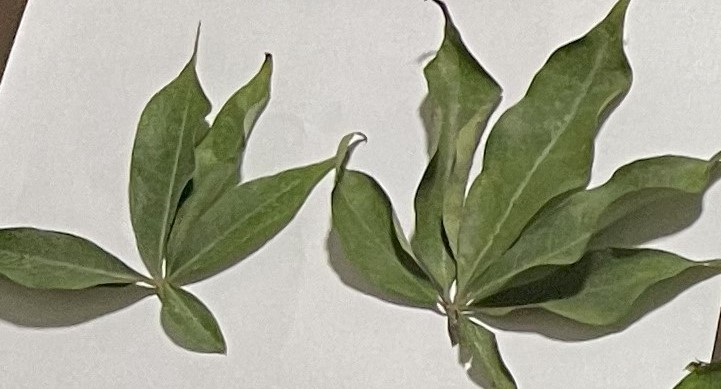 | 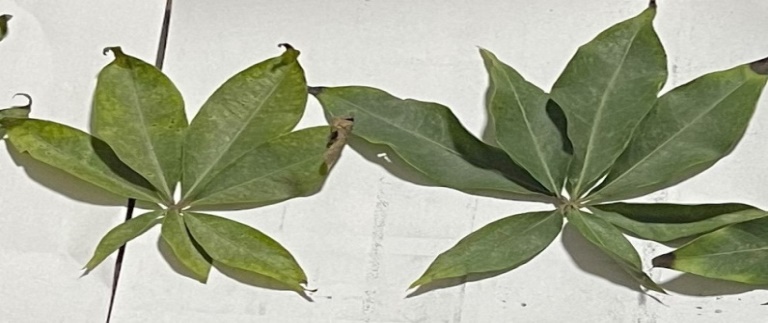 |
| TSW 75%: 0.0 mM GB- TSW 75%: 50.0 mM GB | ADW 75%: 0.0 mM GB- ADW 75%: 50.0 mM GB |
| 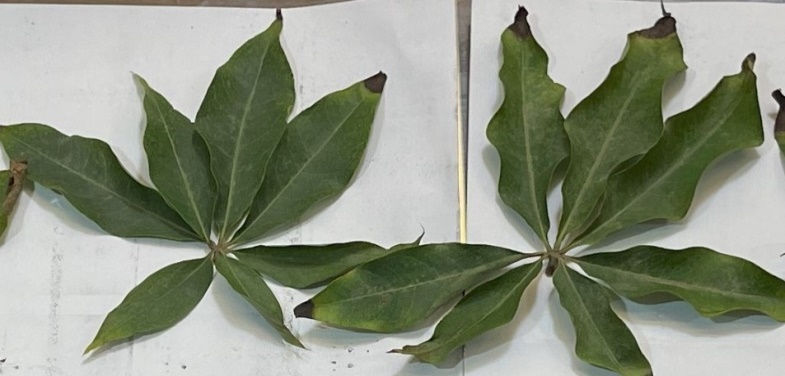 | 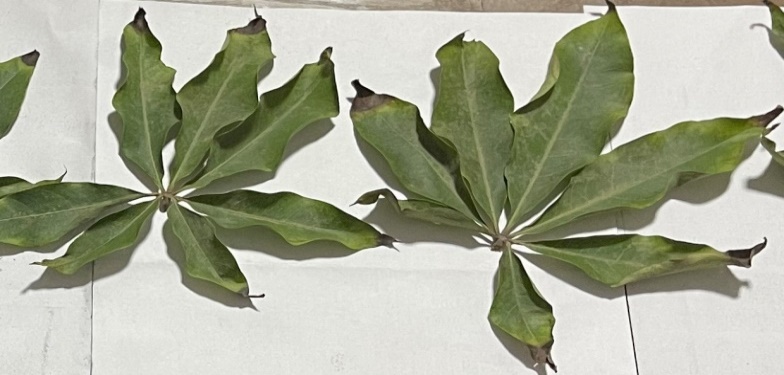 |
| TSW 50%: 0.0 mM GB- TSW 50%: 50.0 mM GB | ADW 50%: 0.0 mM GB- ADW 50%: 50.0 mM GB |
| 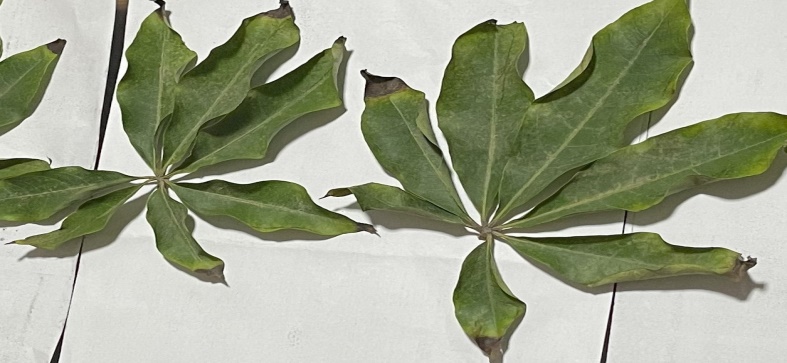 | 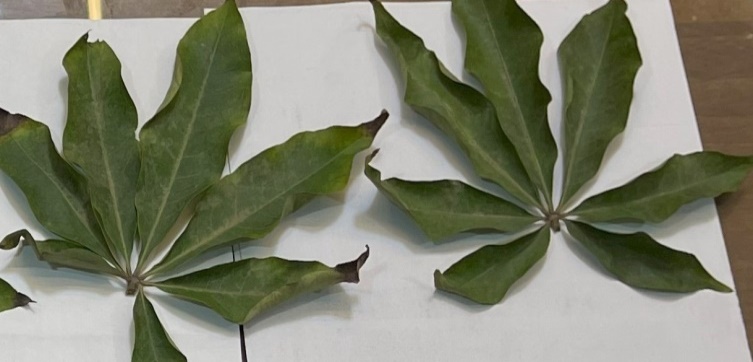 |
| TSW 25%: 0.0 mM GB- TSW 25%: 50.0 mM GB | ADW 25%: 0.0 mM GB- ADW 25%: 50.0 mM GB |
| 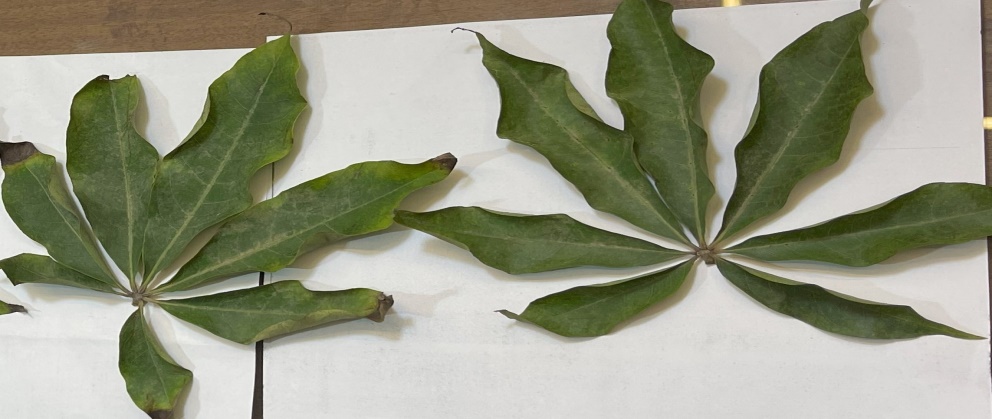 | |
| NIW 100%: 0.0 mM GB -------------NIW 100%: 50.0 mM GB | |
