# Supplementary figures and images for: Response of Kapok seedlings were irrigated with water of different qualities and heavy metal contents for foliar application of antioxidants
Source: BMC Plant Biol. 2025 Jan 3;25:11. doi: 10.1186/s12870-024-05902-y (PMC11697924; doi:10.1186/s12870-024-05902-y)

| 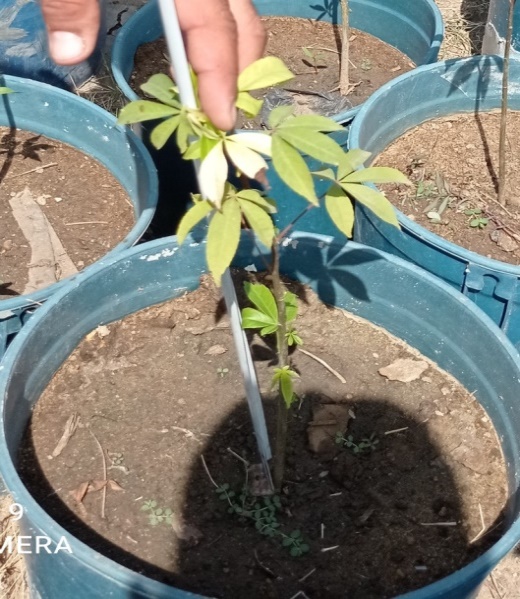 | 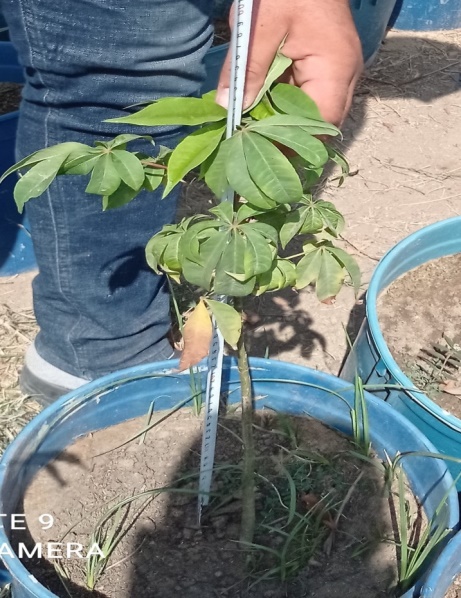 | 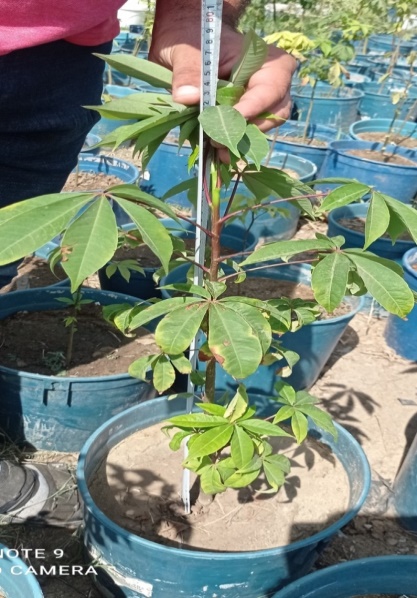 |
| --- | --- | --- |
| **TSW 100%: 0.0 mM GB** | **TSW 75%: 0.0 mM GB** | **TSW 50%: 0.0 mM GB** |
| 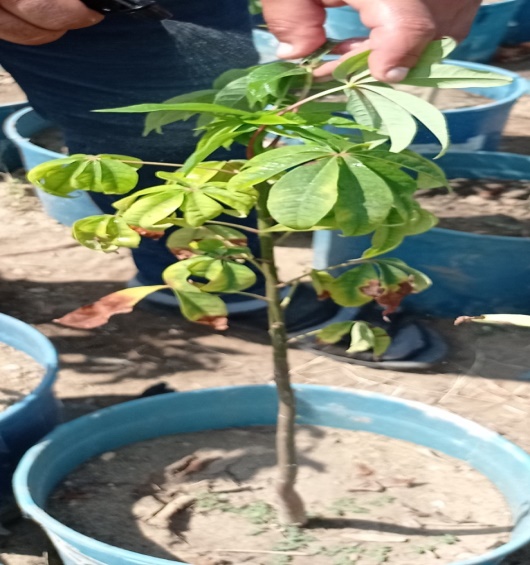 | 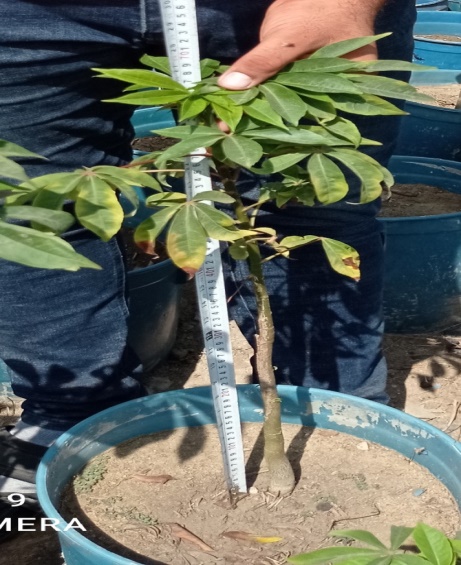 | 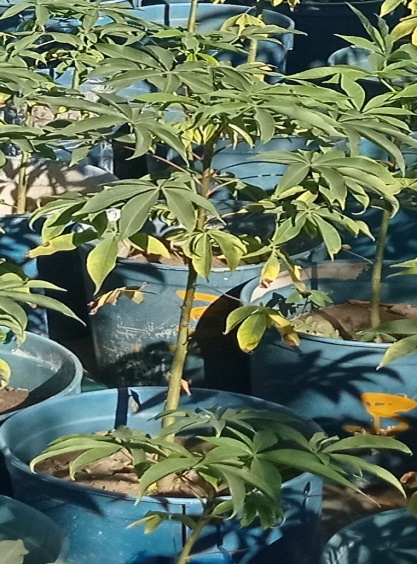 |
| **ADW 100%: 0.0 mM GB** | **ADW 75%: 0.0 mM** | **ADW 50%: 0.0 mM** |
| 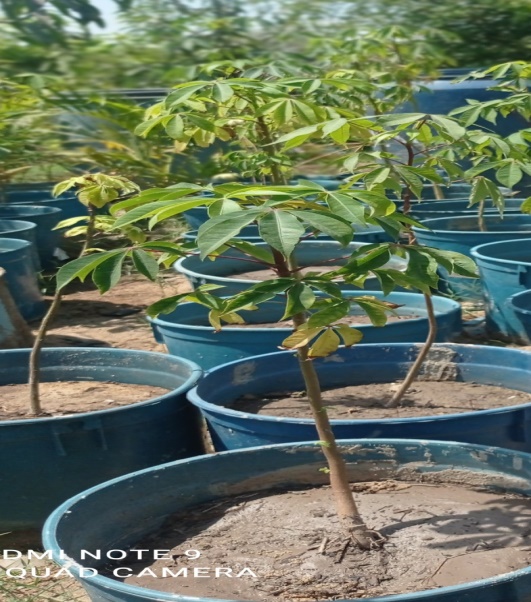 | 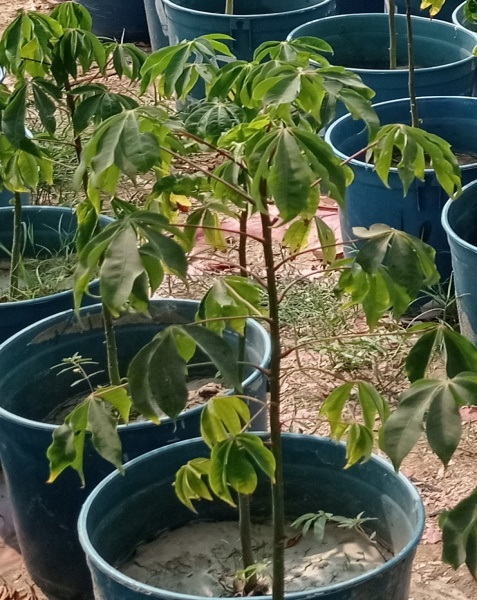 | 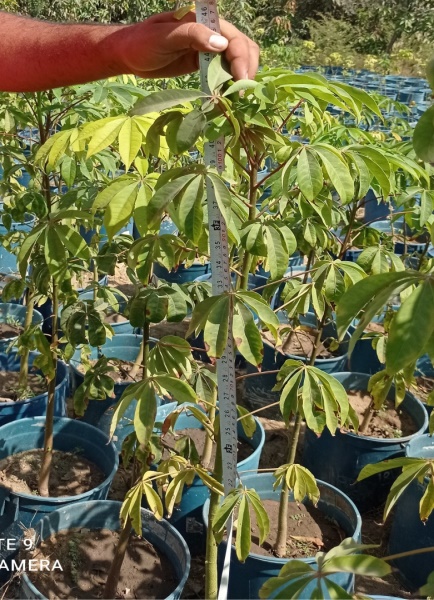 |
| **TSW 25%: 0.0 mM GB** | **ADW 25%: 0.0 mM GB** | **NIW 100%: 0.0 mM GB** |

Supplement: Supplementary file 2 — Supplementary Material 2. [file 12870_2024_5902_MOESM2_ESM.docx]
